# Design of Engineered Cyclodextrin Derivatives for Spontaneous Coating of Highly Porous Metal-Organic Framework Nanoparticles in Aqueous Media

**DOI:** 10.3390/nano9081103

**Published:** 2019-08-01

**Authors:** Giovanna Cutrone, Xue Li, Juan M. Casas-Solvas, Mario Menendez-Miranda, Jingwen Qiu, Gábor Benkovics, Doru Constantin, Milo Malanga, Borja Moreira-Alvarez, José M. Costa-Fernandez, Luis García-Fuentes, Ruxandra Gref, Antonio Vargas-Berenguel

**Affiliations:** 1 Department of Chemistry and Physics, University of Almería, Crta. de Sacramento s/n, E-04120 Almería, Spain; 2 Institut des Sciences Moléculaires d’Orsay, UMR CNRS 8214, Université Paris-Sud, Université Paris Saclay, 91400 Orsay, France; 3 CycloLab R&D Ltd., Illatos út 7, H-1097 Budapest, Hungary; 4 Laboratoire de Physique des Solides, UMR 8502, Université Paris-Sud, 91405 Orsay, France; 5 Department of Physical and Analytical Chemistry, University of Oviedo, Julián Clavería 8, 33006 Oviedo, Spain

**Keywords:** metal-organic frameworks, MIL-100(Fe), β-cyclodextrin, mannose, molecular recognition, “stealth” effect, multivalent effect, isothermal titration calorimetry, macrophage

## Abstract

Nanosized metal-organic frameworks (nanoMOFs) MIL-100(Fe) are highly porous and biodegradable materials that have emerged as promising drug nanocarriers. A challenging issue concerns their surface functionalization in order to evade the immune system and to provide molecular recognition ability, so that they can be used for specific targeting. A convenient method for their coating with tetraethylene glycol, polyethylene glycol, and mannose residues is reported herein. The method consists of the organic solvent-free self-assembly on the nanoMOFs of building blocks based on β-cyclodextrin facially derivatized with the referred functional moieties, and multiple phosphate groups to anchor to the nanoparticles’ surface. The coating of nanoMOFs with cyclodextrin phosphate without further functional groups led to a significant decrease of macrophage uptake, slightly improved by polyethylene glycol or mannose-containing cyclodextrin phosphate coating. More notably, nanoMOFs modified with tetraethylene glycol-containing cyclodextrin phosphate displayed the most efficient “stealth” effect. Mannose-coated nanoMOFs displayed a remarkably enhanced binding affinity towards a specific mannose receptor, such as Concanavalin A, due to the multivalent display of the monosaccharide, as well as reduced macrophage internalization. Coating with tetraethylente glycol of nanoMOFs after loading with doxorubicin is also described. Therefore, phosphorylated cyclodextrins offer a versatile platform to coat nanoMOFs in an organic solvent-free, one step manner, providing them with new biorecognition and/or “stealth” properties.

## 1. Introduction

Metal-organic frameworks (MOFs) are currently among the most versatile materials, with crystalline porous structures built using organic linkers and metal cations. Since they were discovered [1], their applications were focused on biosensing, gas storage, separation, heterogeneous catalysis, and imaging [2,3,4,5,6,7,8]. More recently, their innovative biomedical applications as potential drug delivery nanocarriers are gaining increasing interest for the treatment of a variety of diseases [9,10,11,12]. These materials allow tuning pore sizes and shapes by varying both metal cations and organic linkers, and thus drug interactions with the matrix can be optimized [13]. The opportunity to confer ad hoc chemical and structural features in addition to the peculiar properties of the internal porous volume make MOFs promising drug delivery systems (DDS) for a large variety of guest molecules [14].

Among the large MOFs family, nanosized metal-organic frameworks MIL-100(Fe) (MIL standing for materials of Institute Lavoisier) are a biodegradable material easily synthesized by a “green” microwave assisted hydrothermal method, made of spontaneous coordination between iron(III) cations and trimesate linkers (1,3,5-benzene tricarboxylate) generating a porous architecture. The structure is accessible through mesoporous windows of two different sizes (5.6 and 8.6 Å), and forms internal cages of 24 and 29 Å available for drug incorporation [15]. MIL-100(Fe) nanoMOFs could entrap a wide range of therapeutic molecules providing high loadings up to 20–70 wt% [9], and were well tolerated in vivo in rats since doses up to 220 mg Kg^−1^ did not trigger any toxicity signs up to one week after injection [11,12,16]. Although iron concentration increased in both liver and spleen upon administration, levels remained far from those able to cause liver malfunction, and iron excess was excreted by urine and feces after 15 days [16]. However, appropriate coating of the nanoMOF surface is desirable for several reasons: (i) An adequate coating may overcome nanoMOFs’ tendency to aggregate in aqueous media thus providing colloidal stability to the system; (ii) it could control and modulate drug release; and (iii) it could confer the nanoMOFs with biorecognition and targeting abilities to reach their biological targets through recognition of specific receptors. In order to achieve these goals, one strategy is to employ a noncovalent coating using saccharide derivatives that are large enough to avoid penetration within the nanoMOF’s pores while conferring colloidal stability and modulated drug release. Recently, we have reported a postsynthetic strategy using phosphorylated β-cyclodextrin (β-CD) building-blocks for the preparation of a biocompatible nanoMOFs system [15,17]. Noncovalent coatings were developed by attaching phosphate groups to the macrocycle that subsequently bind the metallic sites on the nanoparticle surface. Advantageously, the procedure was carried out directly in water in one step without using any toxic additives and led to coatings stable enough under physiological conditions. β-CD was chosen because its dimensions are, as needed, large enough to avoid penetration within the MIL-100(Fe) nanoMOFs pores [18], and their numerous free OH are very suitable for introducing functional groups in the primary or/and secondary rims. In addition, β-CD coatings were able to improve suspension stability and could be further functionalized with fluorescent dyes.

Additionally, application of nanoparticles in biological media is strongly affected by their interaction with proteins from the medium. When nanoparticles are exposed to a biological environment, a fast adsorption of proteins onto their surfaces takes place, thus forming a NP-protein complex corona. This so-called “protein corona” modifies the nanoparticles “identity” and triggers various biological processes towards the nanoparticles including blood clearance and immunity response [19]. A potential drug nanocarrier must avoid rapid clearance during the circulation time and should prevent fast cellular uptake. In this regard, long chain polyethylene glycol (PEG) shells are used as a molecular barrier that shields nanoparticles against protein adsorption and fast recognition by the immune system, as it is proved that PEG has one of the lowest levels of protein absorption among all known polymers [20]. Such PEG brushes confer “stealth” properties to the drug delivery systems and lead to a decrease in cellular uptake. However, whereas PEG surface modification has been widely documented in the case of liposomes and polymeric nanoparticles, only a few attempts have been made so far to modify MIL-100(Fe) nanoMOFs with PEG [15,21].

A challenging issue concerning the development of MOF-based nanocarriers is their surface functionalization with molecular recognition properties, so they can be used for specific targeting. In this regard, biomolecules involved in biological recognition processes such as carbohydrates present several advantages over other biomolecules [22,23]. In addition to contributing to the nanoparticle biocompatibility and stability in aqueous media preventing aggregation, they are available on a large scale and relatively easy to chemically modify. Furthermore, they have been proven to provide “stealth” properties comparable to those conferred by PEGylation, while barely affecting the molecular recognition properties [24,25]. However, the interaction between carbohydrates and their specific protein receptors, called lectins, are typically very weak when compared with other biological binding events [26]. Nature uses a well-known strategy, named multivalent effect, to increase this low affinity by gathering multiple copies of sugars to achieve a global binding potency toward their receptors higher than that for the sum of the monovalent interactions [27]. Inspired by this natural approach, a vast number of structures decorated with multiple carbohydrate moieties have been tested as potential theranostic agents in biosensing and biomedicine [22,28,29].

Lectins are overexpressed in some tumor cells [30,31]. Thus, decorating nanoparticles of different types with carbohydrates is an efficient approach to increase the local concentration of anticancer drugs near tumor cells, facilitating specific cellular uptake through the specific binding between the carbohydrate ligand and the overexpressed specific lectin [32,33]. Among this family of proteins, mannose-binding lectins (MBLs) are overexpressed by many human cancer cells and may be used as therapeutic targets [30]. Mannose has been used to target cells of the immune system such as macrophages, dendritic cells, or Langerhans cells, as well as pathogenic microorganisms [34,35,36] and cancer cells including retina, breast, and prostate, due to its high specificity to lectin receptors [37,38,39]. Moreover, it has been recently reported that PEGylated silicon nanoparticles (SiNPs) anchored with mannose had a better “stealth” effect compared to PEGylated SiNPs in vivo [24]. Furthermore, hydroxyethyl starch nanocarriers PEGylated and mannose-functionalized on the outer PEG layer not only showed an efficient “stealth” effect, but also that the targeting moieties were accessible to the biological receptors [25]. However, to the best of our knowledge, only a few examples of nanoMOFs functionalized with mannose have previously been reported by some of us [15,17].

In this context, we describe a convenient preparation of tetraethylene glycol (TEG)-, PEG-, and mannose-surface functionalized MIL-100(Fe) nanoMOFs. The preparation involved, first, the synthesis of a series of phosphorylated β-CD scaffolds displaying motifs of mannose and/or TEG or PEG (average MW ~2000) as coating building blocks, followed by a postsynthetic modification of MIL-100(Fe) nanoMOFs by spontaneous self-assembly of the building blocks on their surfaces. We compare the “stealthy” capacity of the surface-modified nanoMOFs to reduce the cellular uptake and to enhance the retention time, avoiding capture by phagocytic cells. Mannosylated nanoparticles exhibit enhanced binding affinity towards a model mannose-specific lectin concanavalin A (ConA) due to the globular multivalent display of the mannose moieties on their surface, much like the glycocalyx [40]. Moreover, we demonstrate that the coating method can be applied to drug-loaded MIL-100(Fe) nanoMOFs, using anticancer drug doxorubicin (DOX).

## 2. Materials and Methods

### 2.1. Materials

Thin layer chromatography (TLC) was performed on Merck silica gel 60 F_254_ aluminum sheets and developed by UV–vis light, iodine, 5% *v*/*v* sulfuric acid in ethanol, and/or 1% *w*/*v* potassium permanganate in aqueous solution containing 0.1% *w*/*v* sodium hydroxide and 7% *w*/*v* potassium carbonate, depending on the case. Flash column chromatography was performed on Merck silica gel (230–400 mesh, ASTM). Melting points were measured on a Büchi B-450 melting point apparatus and are uncorrected. Optical rotations were recorded on a Jasco P-1030 polarimeter at room temperature. [α]_D_^25^ values are given in 10^−1^ deg cm^−1^ g^−1^. Infrared spectra were recorded on a Bruker Alpha FTIR equipped with a Bruker universal ATR sampling accessory. ^1^H, ^13^C, and 2D NMR spectra were recorded on a Bruker Avance III HD 600 MHz spectrometer equipped with a QCI ^1^H/^13^C/^15^N/^31^P proton-optimized quadrupole inverse cryoprobe with ^1^H and ^13^C cryochannels, a Bruker Avance III HD 500 MHz spectrometer equipped with an inverse TBI ^1^H/^31^P/BB probe, or a Bruker Nanobay Avance III HD 300 MHz spectrometer equipped with a QNP ^1^H/^13^C/^19^F/^31^P probe, depending on the sample. Standard Bruker software was used for acquisition and processing routines. Chemical shifts (δ) are given in parts per million (ppm) and referenced to internal tetramethylsilane (TMS) signal (δ_H_, δ_C_ 0.00). *J* values are given in hertz (Hz). MALDI-TOF mass spectra were recorded on a 4800 Plus AB SCIEX spectrometer with 2,5-dihydroxybenzoic acid (DHB) as the matrix. ESI-TOF mass spectra were measured on an Agilent LC/MSD-TOF spectrometer. Syringe filtering was conducted using nylon 0.45 μm Milipore Millex^®^ syringe-driven filter units. Dialysis was performed using Thermo Scientific^TM^ Slide-A-Lyzer^TM^ G2 2000 MWCO Dialysis Cassette. A Hanna HI 98192 EC/TDS/NaCl/Resistivity meter was employed to monitor dialysate solutions conductivity during dialysis. Transmission electron microscope (TEM) images were acquired on a JEOL 1400 microscope working at 120 kV. Dynamic light scattering (DLS) measurements were performed on a Malvern Zetasizer Nano-ZS analyzer. Nitrogen sorption experiments were conducted on a Micromeritics ASAP 2020 equipment. Inductively coupled plasma mass spectrometry (ICP-MS) analysis was performed on an Agilent 8800 instrument equipped with a triple quadrupole. Isothermal titration calorimetry (ITC) experiments were conducted on an ultrasensitive Microcal VP-ITC calorimeter.

Acetic anhydride (Panreac, purum), per(6-azido-6-deoxy)-β-CD **1** (CycloLab L&D Ltd., Budapest, Hungary), copper(I) bromide (Sigma-Aldrich, St. Louis, MI, USA, ≥99.9%), copper(I) iodide (Aldrich, 98%), anhydrous copper(II) sulphate (Fluka, 98%), (+)-sodium l-ascorbate (NaAsc, Sigma, BioXtra, ≥99%), phosphorus pentoxide (Panreac, purum), 1-methoxypentatetraconta(ethyle- ne glycol) (Aldrich, M_n_ ~2000), 4-dimethylaminopyridine (DMAP, Fluka, ≥98%), methanesulfonyl chloride (Fluka, ≥99%), *tert*-butyldimethylsilyl chloride (Sigma-Aldrich, Steinheim, Germany, 97%), doxorubicin (DOX, Sigma-Aldrich, St. Louis, MI, USA, 98%), and β-CD phosphate sodium salt (CD-P, Cyclolab L&D Ltd., Budapest, Hugary, DS 2–6), were purchased from commercial sources and used without further purification unless otherwise indicated. β-CD (CycloLab L&D Ltd., Budapest, Hungary) was dried at 50 °C in vacuum in the presence of P_2_O_5_ until it reached a constant weight before using. Propargyl 2,3,4,6-tetra-*O*-acetyl-α-d-mannopyranoside **2** was prepared as described in literature [41,42] with small modifications. Specifically, purification of the compound after deacetylation was carried out by flash column chromatography using EtOAc-MeOH 6:1 as eluent. NMR data for this compound in D_2_O completely agreed with that described by van der Peet et al. [43]. 1-Propargyloxy-3,6,9-trioxa-undecane-11-ol [44,45], 3,6,9,12-tetraoxapentadec-14-ynyl 2,3,4,6-tetra-*O*-acetyl-α-d-mannopyranoside **4** [46], heptakis(6-*O*-*tert*-butyldimethylsilyl-2,3-di-*O*- propargyl)cyclomaltoheptaose **12** [47], and heptakis(2,3-di-*O*-propargyl)cyclomaltoheptaose **13** [47] were prepared as described in literature. Pyridine (Panreac, purum), trimethylamine (Sigma-Aldrich, ≥99%), and organic solvents were dried according to literature procedures [48]. Dry DMF (AcroSeal, 99.8%, over molecular sieves) was purchased from Acros.

Iron (III) chloride hexahydrate (Alfa Aesar, 98%), 1,3,5-benzenetricarboxylic acid (BTC, Sigma-Aldrich, St. Louis, MI, USA, 95%), and absolute ethanol (Carlo Erba, 99%,) were used for the synthesis of nanoMOFs. CD-P was used for the coating of nanoMOFs along with the synthesized CD phosphate salt derivatives **8**–**10** and **16**. Dulbecco’s PBS solution was used for nanoMOFs Zeta potential (ZP) measurements. Stability studies were performed in a complete cell culture medium (DMEM, in Dulbecco’s Modified Eagle’s Medium (Thermo Fischer, Waltham, MA, USA) complemented with 10% FBS, 1% penicillin/streptomycin (100 mg/mL), and 1% l-glutamine). Deionized MilliQ water was obtained from a Millipore apparatus with a 0.22 μm filter.

### 2.2. Synthesis of 1-*O*-propargyl-13-*O*-acetyl-1,4,7,10,13-pentaoxatridecane (***3***)

Acetic anhydride (3 mL, 0.032 mol) was added at 0 °C to a solution of 1-propargyloxy-3,6,9-tri- oxa-undecane-11-ol (1.228 g, 5.287 mmol) in dry pyridine (6 mL, 0.074 mol) and left at room temperature overnight. Solvent was rotary evaporated under high vacuum and the crude was purified by flash column chromatography using 2:3→1:2 Hexane-EtOAc as eluent to give compound **3** (1.370 g, 4.994 mmol, 94%) as a colorless oil. R_f_ = 0.6 (1:2 Hexane-EtOAc); IR ν/cm^−1^ 2872, 2116, 1733, 1238, 1095, 1049, 955, 919, 729; ^1^H-NMR (500 MHz, CDCl_3_), δ (ppm): 4.23–4.21 (m, 2H, H-12), 4.20 (d, 2H, *J* = 2.4 Hz, OCH_2_C≡), 3.71–3.68 (m, 6H, H-2,3,11), 3.67 (s, 4H, OC*H*_2_C*H*_2_O), 3.66 (s, 4H, OC*H*_2_C*H*_2_O), 2.43 (t, 1H, *J* = 2.4 Hz, ≡CH), 2.08 (s, 3H, CH_3_); ^13^C-RMN (125 MHz, CDCl_3_), δ (ppm): 171.1 (CO), 79.7 (C≡), 74.6 (≡CH), 70.7–70.5 (C-3,5,6,8,9), 69.2–69.1 (C-2,11), 63.7 (C-12), 58.5 (O*C*H_2_C≡), 21.0 (CH_3_); ESI-TOF-MS *m*/*z* calcd. for C_13_H_26_NO_6_ 292.1756, found 292.1755 (M + NH_4_)^+^; calcd. for C_13_H_22_NaO_6_ 297.1309, found 297.1309 (M + Na)^+^.

### 2.3. Synthesis of Heptakis{6-deoxy-6-[4′-(2″,3″,4″,6″-tetra-*O*-acetyl-α-d-mannopyranosyloxymethyl)-1*H*- 1,2,3-triazol-1′-yl]}cyclomaltoheptaose (***5***)

A suspension of CuBr (426 mg, 2.97 mmol) in H_2_O (45 mL) was added to a preheated (100 °C) solution of per(6-azido-6-deoxy)-β-CD **1** (200 mg, 0.142 mmol) and propargyl 2,3,4,6-tetra-*O*-acetyl- α-d-mannopyranose **2** (47 mg, 0.213 mmol) in DMF (15 mL). The mixture was stirred for 1 h at 100 °C. The solvent was evaporated under high vacuum and the residue was purified by a flash column chromatography using 10:1→5:1 CH_3_CN-H_2_O. Subsequently, the product was passed through a short pad of silica gel using 10:2:1 CH_3_CN-H_2_O-(30% *v*/*v* aq. NH_3_) as eluent. The collected fractions were rapidly neutralized to pH ~7.0 by addition of 1 N HCl and extracted with CH_2_Cl_2_ (100 mL) to prevent deacetylation. The organic layer was washed with H_2_O (2 × 100 mL), dried on Na_2_SO_4_, and evaporated to yield compound **5** (2.6 g, 0.655 mmol, 86%) as a pale yellow solid: R_f_ = 0.2 [25:1:5 CH_3_CN-H_2_O-(30% *v*/*v* aq. NH_3_)]; mp 96 °C; [α]_D_^25^ +70° (*c* 0.5, CH_2_Cl_2_); IR ν/cm^−1^ 3418, 2934, 1748, 1372, 1230, 1048; ^1^H-NMR (500 MHz, DMSO-*d*_6_, 80 °C), δ (ppm): 7.96 (s, 7H, H-5′), 5.66–5.65 (m, 14H, OH), 5.13–5.10 (m, 21H, H-1,3″,4″), 5.06 (dd, 7H, ^3^*J*_1″,2″_ = 1.6 Hz, ^3^*J*_2″,3″_ = 3.0 Hz, H-2″), 4.91 (d, 7H, ^3^*J*_1″,2″_ = 1.6, H-1″), 4.65 (d, 7H, ^2^*J* = 12.4 Hz, OC*H*^a^-C_2_HN_3_), 4.57 (dd, 7H, ^2^*J*_6a,6b_ = 14.1 Hz, ^3^*J*_5,6a_ = 2.5 Hz, H-6^a^), 4.48 (d, 7H, ^2^*J* = 12.4 Hz, OC*H*^b^-C_2_HN_3_), 4.46 (dd, 7H, ^2^*J*_6a,6b_ = 14.1 Hz, ^3^*J*_5,6b_ = 4.8 Hz, H-6^b^), 4.16 (dd, ^2^*J*_6″a,6″b_ = 12.2 Hz, ^3^*J*_5″,6″a_ = 5.1 Hz, H-6″^a^), 4.13 (m, 7H, H-5), 4.07 (dd, 7H, ^2^*J*_6″a,6″b_ = 12.2 Hz, ^3^*J*_5″,6″b_ = 2.8 Hz, H-6″^b^), 3.99 (ddd, 7H, ^3^*J*_4″,5″_ = 9.7 Hz, ^3^*J*_5″,6″a_ = 5.1 Hz, ^3^*J*_5″,6″b_ = 2.8 Hz, H-5″), 3.76 (td, 7H, ^3^*J* = 9.5 Hz, ^3^*J* = 2.3 Hz, H-3), 3.33–3.29 (m, 7H, H-2), 3.26 (t, 7H, *J* = 9.2 Hz, H-4), 2.07 (s, 21H, CH_3_), 2.01 (s, 21H, CH_3_), 1.99 (s, 21H, CH_3_), 1.89 (s, 21H, CH_3_); ^13^C-NMR (125 MHz, DMSO-*d*_6_, 80 °C), δ (ppm): 169.4, 168.9, 168.8, 168.7 (CO), 141.9 (C-4′), 125.7 (C-5′), 101.4 (C-1), 95.7 (C-1″), 82.2 (C-4), 72.0 (C-3), 71.6 (C-2), 69.1 (C-5), 68.5 (C-3″), 68.4 (C-2″), 67.8 (C-5″), 65.5 (C-4″), 61.6 (C-6″), 59.5 (O*C*H_2_-C_2_HN_3_), 49.1 (C-6), 19.9, 19.8, 19.7, 19.6 (CH_3_). MALDI-TOF-MS *m*/*z* calcd. for C_161_H_217_N_21_O_98_Na^+^ 4035.3, found 4035.9 (M+Na)^+^.

### 2.4. Synthesis of Heptakis(6-deoxy-6-{4′-[14″-*O*-acetyl-(2″,5″,8″,11″,14″-pentaoxatetradecyl)]-1*H*-1,2,3-tri- azol-1′-yl})cyclomaltoheptose (***6***)

A suspension of CuI (300 mg, 1.58 mmol) in H_2_O (30 mL) was added to a preheated (100 °C) solution of per(6-azido-6-deoxy)-β-CD **1** (413 mg, 0.32 mmol) and 1-*O*-propargyl-13-*O*-acetyl- 1,4,7,10,13-pentaoxatridecane **3** (1.73 g, 6.3 mmol) in DMF (30 mL). The mixture was stirred for 6 h at 100 °C. The reaction was followed by TLC using 5:1 1,4-dioxane-(30% *v*/*v* aq. NH_3_) as eluent until only one spot at R_f_ = 0.4 was observed. The solvent was evaporated under high vacuum and the residue was purified by a flash column chromatography using 10:1:2 CH_3_CN-H_2_O-(30% *v*/*v* aq. NH_3_) as eluent. The collected fractions were rapidly neutralized to pH ~7.0 by addition of 1 N HCl and extracted with CH_2_Cl_2_ (3 × 100 mL) to prevent deacetylation. The organic layer was washed with H_2_O (1 × 50 mL), dried on MgSO_4_, filtered, and rotary evaporated to yield compound **6** (418 mg, 0.129 mmol, 41%) as a sticky pale yellow solid: R_f_ = 0.4 [5:1 1,4-dioxane-(30% *v*/*v* aq. NH_3_)]; [α]_D_^25^ +8° (*c* 0.25, CH_2_Cl_2_); IR ν/cm^−1^ 3354, 2873, 1734, 1237, 1091, 1045; ^1^H-NMR (500 MHz, DMSO-*d*_6_, 80 °C), δ (ppm): 7.86 (s, 7H, H-5′), 5.64 (bs, 14H, OH-2,3), 5.08 (d, 7H, ^3^*J*_1,2_ = 3.2 Hz, H-1), 4.45 (dd, 7H, ^2^*J*_6a,6b_ = 14.5 Hz, ^3^*J*_5,6a_ = 2.6 Hz, H-6^a^), 4.40 (d, 7H, ^2^*J* = 12.4 Hz, H-1″^a^), 4.34 (d, 7H, ^2^*J* = 12.4 Hz, H-1″^b^), 4.32 (dd, 7H, ^2^*J*_6a,6b_ = 14.5 Hz, ^3^*J*_5,6b_ = 7.9 Hz, H-6^b^), 4.11 (t, 14H, *J* = 5.0 Hz, H-13″), 4.10–4.07 (m, 7H, H-5), 3.75 (t, 7H, *J* = 9.2 Hz, H-3), 3.60 (t, 14H, *J* = 5.0 Hz, H-12″), 3.55–3.49 (m, 84H, H-2″,3″,6″,7″,9″,10″), 3.31 (dd, 7H, ^3^*J*_2,3_ = 9.6 Hz, ^3^*J*_1,2_ = 3.2 Hz, H-2), 3.28 (t, 7H, *J* = 9.4 Hz, H-4), 2.00 (s, 21H, CH_3_); ^13^C-NMR (125 MHz, DMSO-*d*_6_, 80 °C), δ (ppm): 169.6 (CO), 143.4 (C-4′), 124.6 (C-5′), 101.3 (C-1), 82.4 (C-4), 72.0 (C-3), 71.6 (C-2), 69.4–69.2 (C-2″,3″,6″,7″,9″,10″), 68.7 (C-5), 67.9 (C-12″), 63.1 (C-1″), 62.6 (C-13″), 49.1 (C-6), 20.0 (CH_3_); MALDI-TOF-MS *m*/*z* calcd. for C_133_H_217_N_21_O_89_Na^+^ 3252.4, found 3252.7 (M + Na)^+^.

### 2.5. Synthesis of Heptakis(6-deoxy-6-{4′-[14″-*O*-(2‴,3‴,4‴,6‴-tetra-*O*-acetyl-α-d-mannopyranosyl)-2″, 5″,8″,11″,14″-pentaoxatetradecyl]-1*H*-1,2,3-triazol-1′-yl})cyclomaltoheptose (***7***)

A suspension of CuSO_4_ (164 mg, 1.02 mmol) and sodium ascorbate (220 mg, 1.11 mmol) in H_2_O (12 mL) was added to a preheated (65 °C) solution of per(6-azido-6-deoxy)-β-CD **1** (362 mg, 0.28 mmol) and compound **4** (1.4 g, 2.5 mmol) in DMF (25 mL). The mixture was stirred for 3 h at 100 °C. The reaction was followed by TLC using 5:1 1,4-dioxane-(30% *v*/*v* aq. NH_3_) as eluent. The solvent was evaporated under high vacuum and the residue passed through a short pad of silica gel using 20:6:1 CH_3_CN-H_2_O-(30% *v*/*v* aq. NH_3_) as eluent. The solution was freeze-dried and the resulting material was further purified by a flash column chromatography using 2:2:1:0→20:20:10:1 CH_2_Cl_2_-CH_3_CN-EtOH-H_2_O as eluent to yield compound **7** (860 mg, 0.164 mmol, 60%) as a sticky pale yellow solid after freeze-drying: R_f_ = 0.4 (5:1 1,4-dioxane-(30% *v*/*v* aq. NH_3_)); [α]_D_^25^ +27° (*c* 0.5, CH_2_Cl_2_); IR ν/cm^−1^ 2877, 1743, 1369, 1220, 1077, 1042, 732; ^1^H-NMR (500 MHz, DMSO-*d*_6_, 80 °C), δ (ppm): 7.86 (s, 7H, H-5′), 5.65–5.64 (m, 14H, OH-2,3), 5.17 (dd, 7H, ^3^*J*_3‴,4‴_ = 10.1 Hz, ^3^*J*_2‴,3‴_ = 3.3 Hz, H-3‴), 5.12 (dd, 7H, ^3^*J*_2‴,3‴_ = 3.3 Hz, ^3^*J*_1‴,2‴_ = 1.4 Hz, H-2‴), 5.11 (t, 7H, *J* = 9.8 Hz, H-4‴), 5.09 (d, 7H, ^3^*J*_1,2_ = 3.2 Hz, H-1), 4.90 (d, 7H, ^3^*J*_1‴,2‴_ = 1.4 Hz, H-1‴), 4.47 (dd, 7H, ^2^*J*_6a,6b_ = 14.7 Hz, ^3^*J*_5,6a_ = 2.7 Hz, H-6^a^), 4.40 (d, 7H, ^2^*J*_1″a,1″b_ = 12.4 Hz, H-1″^a^), 4.35 (bd, 7H, *J* = 14.1 Hz, H-6^b^), 4.34 (d, 7H, ^2^*J*_1″a,1″b_ = 12.4 Hz, H-1″^b^), 4.15 (dd, 7H, ^2^*J*_6‴a,6‴b_ = 12.2 Hz, ^3^*J*_5‴,6‴a’_ = 5.2 Hz, H-6‴^a^), 4.11–4.09 (m, 7H, H-5), 4.08 (dd, 7H, ^2^*J*_6‴a,6‴b_ = 12.2 Hz, ^3^*J*_5‴,6‴b_ = 2.9 Hz, H-6‴^b^), 4.01 (ddd, 7H, ^3^*J*_4‴,5‴_ = 9.6 Hz, ^3^*J*_5‴,6‴a_ = 5.2 Hz, ^3^*J*_5‴,6‴b_ = 2.0 Hz, H-5‴), 3.77–3.75 (m, 7H, H-3), 3.74 (t, 7H, *J* = 4.5 Hz, H-13″^a^), 3.66 (dd, 7H, ^2^*J* = 9.6 Hz, ^3^*J* = 5.1 Hz, H-13″^b^), 3.62 (dd, 14H, *J* = 9.6 Hz, *J* = 5.0 Hz, H-12″), 3.57–3.48 (m, 84H, H-3″,4″,6″,7″,9″11″), 3.34–3.29 (m, 7H, H-2), 3.27 (t, 7H, *J* = 9.4 Hz, H-4), 2.10 (s, 21H, CH_3_), 2.01 (s, 21H, CH_3_), 2.00 (s, 21H, CH_3_), 1.93 (s, 21H, CH_3_); ^13^C-NMR (125 MHz, DMSO-*d*_6_, 80 °C), δ (ppm): 169.3 (CO), 168.9 (CO), 168.8 (CO), 167.8 (CO), 143.4 (C-4′), 124.7 (C-5′), 101.3 (C-1), 96.5 (C-1‴), 82.4 (C-4), 72.0 (C-3), 71.6 (C-2), 69.5–69.4 (O*C*H_2_*C*H_2_O), 69.3 (C-5), 68.9 (C-12″), 68.7 (O*C*H_2_*C*H_2_O), 68.6 (C-2‴), 68.4 (C-3‴), 67.7 (C-5‴), 66.4 (C-13″), 65.6 (C-4‴), 63.1 (C-1″), 61.8 (C-6‴), 49.1 (C-6), 19.9 (CH_3_), 19.8 (CH_3_), 19.7 (CH_3_), 19.6 (CH_3_); MALDI-TOF-MS *m*/*z* calcd. for C_217_H_329_N_21_O_126_ 5245.00, found 5245.7 (M)^+^; calcd. for C_217_H_329_N_21_O_126_Na^+^ 5268.0, found 5267.6 (M + Na)^+^.

### 2.6. Synthesis of Heptakis{6-deoxy-6-[4′-(α-d-mannopyranosyloxymethyl)-1*H*-1,2,3-triazol-1′-yl]}cyclomal- toheptaose Phosphate Sodium Salt (***8***)

P_2_O_5_ (2.7 g, 19 mmol) was suspended in dry DMF (40 mL) and sonicated for 30 min, then compound **5** (1.25 g, 0.311 mmol) was added and the mixture was stirred at 40 °C until no starting material was detectable (after 4 h) by TLC [2:1:2 CH_3_CN-H_2_O-(30% *v*/*v* aq. NH_3_)]. The mixture was then stirred for 12 h at room temperature at pH 11–13, which was maintained by addition of 1 M aqueous NaOH as needed. The solution was then neutralized with 5% aqueous HCl and the solvent was rotary evaporated under high vacuum. The residue was dissolved in the minimum amount of H_2_O, syringe filtered (nylon 0.45 μm), and dialyzed (2000 MWCO) against distilled water by changing dialysate solution every 3 h until its conductivity was stable and below 1 μS/cm (5 days for final value of 0.96 μS/cm) to yield **8** (1.3 g) as a white solid after lyophilization: mp 250 °C (dec); IR ν/cm^−1^ 3420, 2933, 2790, 1645, 1255, 1126, 1052, 977, 889, 537; ^1^H-NMR (600 MHz, D_2_O), δ (ppm): 8.12 (bs, H-5′), 5.32–5.21, 4.8–4.11 (overlapped with HDO), 3.83–3.52; ^13^C-NMR (150 MHz, D_2_O), δ (ppm): 143.4 (C-4′), 126.9 (C-5′), 99.3 (C-1″), 72.9, 72.0, 70.4, 69.8, 66.6, 60.8 (C-6″), 59.5 (O*C*H_2_-C_2_HN_3_), 50.5 (C-6); ^13^P-RMN (242.9 MHz, D_2_O), δ (ppm): 0.71 (orthophosphates), −8.38 (pyrophosphates), −21.51–(−23.65) (polyphosphates); HR-ICP-MS: P 16.3%.

### 2.7. Synthesis of Heptakis{6-deoxy-6-[4′-(13″-hydroxy-2″,5″,8″,11″-tetraoxatridecyl)-1*H*-1,2,3-triazol-1′- yl]}cyclomaltoheptose Phosphate Sodium Salt (***9***)

P_2_O_5_ (2.067 g, 14.560 mmol) was suspended in dry DMF (13 mL) and sonicated for 30 min, then compound **6** (847 mg, 0.260 mmol) was added and the mixture was stirred at 40 °C until no starting material was observed (after 4 h) by TLC [5:1 1,4-dioxane-(30% *v*/*v* aq. NH_3_)]. The mixture was then stirred for 12 h at room temperature at pH 11–13, which was maintained by addition of 1 M aqueous NaOH as needed. The solution was then neutralized with 5% aqueous HCl and the solvent rotary evaporated under high vacuum. The residue was dissolved in the minimum amount of H_2_O, syringe filtered (nylon 0.45 μm), and dialyzed (2000 MWCO) against distilled water by changing dialysate solution every 3 h until its conductivity was stable and below 1 μS/cm (5 days for final value of 0.90 μS/cm) to yield **9** (669 mg) as a white solid after lyophilization: mp 160 °C; IR ν/cm^−1^ 3432, 2922, 2877, 1640, 1267, 1090, 946, 522; ^1^H-NMR (600 MHz, D_2_O), δ (ppm): 7.94 (bs, H-5′), 5.11 (bs, H-1), 4.66–3.96 (m, H-1″,3,5,6^a,b^), 3.55–3.46 (m, H-2,3″,4,4″,6″,7″.9″,10″,11″,12″); ^13^C-NMR (150 MHz, D_2_O), δ (ppm): 143.8 (C-4′), 126.8 (C-5′), 99.7 (C-1), 81.7, 79.1, 75.7–73.9, 71.7 (O*C*H_2_*C*H_2_O), 70.2, 69.5–69.0 (O*C*H_2_*C*H_2_O), 63.0 (C-1″), 60.3 (C-13″), 50.4 (C-6); ^13^P-RMN (242.9 MHz, D_2_O), δ (ppm): 0.20 (orthophosphates), −9.69 (pyrophosphates), −21.57–(−23.65) (polyphosphates); HR-ICP-MS: P 15.7%.

### 2.8. Synthesis of Heptakis{6-desoxi-6-{4′-[14″-*O*-(α-d-mannopyranosyl)-2″,5″,8″,11″,14″-pentaoxatetrade- cyl]-1*H*-1,2,3-triazol-1′-yl}}ciclomaltoheptose Phosphate Sodium Salt (***10***)

P_2_O_5_ (510 mg, 3.6 mmol) was suspended in dry DMF (13 mL) and sonicated for 30 min, then compound **7** (356 mg, 0.068 mmol) was added and the mixture was stirred at 40 °C until no starting material was observed (after 5 h) by TLC [5:1 1,4-dioxane-(30% *v*/*v* aq. NH_3_)]. The mixture was then stirred for 12 h at room temperature at pH 11–13, which was maintained by addition of 1 M aqueous NaOH as needed. The solution was then neutralized with 5% aqueous HCl and the solvent rotary evaporated under high vacuum. The residue was dissolved in the minimum amount of H_2_O, syringe filtered (nylon 0.45 μm), and dialyzed (2000 MWCO) against distilled water by changing dialysate solution every 3 h until its conductivity was stable and below 1 μS/cm (5 days for final value of 0.98 μS/cm) to yield **10** (806 mg) as a white solid after lyophilization: mp 110 °C; IR ν/cm^−1^ 3431, 2923, 1643, 1271, 1092, 1030, 975, 529; ^1^H-NMR (600 MHz, D_2_O), δ (ppm): 8.07 (bs, H-5′), 5.23–5.16, 4.55–4.10 (overlapped with HDO), 3.97, 3.88–3.82, 3.78–3.75, 3.72–3.65; ^13^C-NMR (150 MHz, D_2_O), δ (ppm): 143.8 (C-4′), 126.8 (C-5′), 100.0 (C-1,1‴) 79.1, 75.2, 72.7, 71.7, 70.5, 70.0, 69.6, 69.5, 69.0, 67.4 66.7, 66.4 (C-13″), 63.0 (C-1″), 60.9 (C-6‴), 50.3 (C-6); ^13^P-RMN (242.9 MHz, D_2_O), δ (ppm): 0.90 (orthophosphates), −7.09–(−8.06) (pyrophosphates), −21.01–(−23.66) (polyphosphates); HR-ICP-MS: P 19.9%.

### 2.9. Synthesis of Heptakis(6-*O*-*tert*-butyldimethylsilyl)cyclomaltoheptaose (***11***)

Compound **11** was prepared as described in literature [49] with a new purification protocol. Specifically, *tert*-butyldimethylsilyl chloride (5.577 g, 37.002 mmol) was added to a solution of β-CD (5 g, 4.405 mmol) in dry pyridine (80 mL) under nitrogen atmosphere and stirred at room temperature for 12 h. The reaction was followed by TLC (30:5:4 EtOAc–96% *v*/*v* EtOH–H_2_O), which showed three spots at R_f_ = 0.7, 0.6, and 0.3 identified as oversilylated byproducts, derivative **11**, and undersilylated species, respectively. Portions of TBDMSCl (664 mg, 4.405 mmol) were added every 12 h until the most polar product was consumed. Water (500 mL) was added and the resulting solid was filtered, washed with water (250 mL), and azeotropically dried with toluene (3 × 150 mL). The residue was purified by a short (5 cm) flash chromatography using 40:40:20:4 CH_2_Cl_2_–MeCN–96% *v*/*v* EtOH–30% *v*/*v* aqueous NH_3_ as eluent until the TLC spot at R_f_ = 0.73 completely eluted. The eluent was then changed to 40:40:20:4 CH_2_Cl_2_–MeCN–96% *v*/*v* EtOH–H_2_O to yield **2** as a white powder which was dried at 50 °C under high vacuum until constant weight (7.302 g, 3.778 mmol, 86%). NMR data agreed with those previously reported [49]: ^1^H NMR (500 MHz, CDCl_3_), δ (ppm): 6.74 (bs, OH), 5.27 (bs, OH), 4.89 (d, 7H, *J*_1,2_ = 3.5 Hz, H-1), 4.04 (t, 7H, *J* = 9.2 Hz, H-3), 3.90 (dd, *J*_6,6′_ = 11.3 Hz, *J*_5,6_ = 2.9 Hz, H-6), 3.71 (bd, 7H, *J* = 10.5 Hz, H-6′), 3.64 (dd, 7H, *J*_2,3_ = 9.6 Hz, *J*_1,2_ = 3.5 Hz, H-2), 3.62 (bs, 7H, H-5), 3.56 (t, 7H, *J* = 9.2 Hz, H-4), 0.87 (s, 63H, SiC(CH_3_)_3_), 0.04 (s, 21H, SiCH_3_), 0.03 (s, 21H, SiCH_3_); ^13^C NMR (125 MHz, CDCl_3_), δ (ppm): 102.1 (C-1), 81.9 (C-4), 73.7 (C-2), 73.5 (C-3), 72.7 (C-5), 61.8 (C-6), 26.0 (SiC(*C*H_3_)_3_), 18.4 (Si*C*(CH_3_)_3_), −4.9 (SiCH_3_), −5.0 (SiCH_3_).

### 2.10. Synthesis of 1-azido-1-deoxy-ω-*O*-methoxypentatetraconta(ethylene glycol) (***14***)

A solution of 1-methoxypentatetraconta(ethylene glycol) (35 g, 17.375 mmol), DMAP (428 mg, 3.5 mmol), and distilled Et_3_N (5.6 mL, 40.250 mmol) in CH_2_Cl_2_ (40 mL) was cooled to 0 °C under inert atmosphere. MsCl (2.7 mL, 35 mmol) was added dropwise over 15 min and the mixture was stirred at 0 °C for 30 min and overnight at room temperature. The reaction mixture was then diluted with CH_2_Cl_2_ (50 mL) and washed with a 5% *v*/*v* aqueous HCl solution (3 × 50 mL) and brine (50 mL). The organic phase was dried over MgSO_4_, filtered, and concentrated under reduced pressure to dryness. The solid was subsequently dissolved in dry DMF (40 mL) and NaN_3_ (2.276 g, 35 mmol) was added. The mixture was stirred at 60 °C for 24 h before the solvent was rotary evaporated under high vacuum. The residue was suspended in THF (20 mL), sonicated (5 min), and the solid filtered off. The clear organic filtrate was rotary evaporated, and the resulting solid was suspended in Et_2_O (50 mL), sonicated (5 min), and filtered. The solid was dissolved in H_2_O (100 mL) and extracted with CH_2_Cl_2_ (3 × 100 mL). The organic phases were combined, dried (MgSO_4_), rotary evaporated, and the residue dried under vacuum to give compound **14** (26.225 g, 12.858 mmol, 74%) as a slightly yellow powder: FT-IR (KBr) ν/cm^−1^: 2868, 2105, 1093, 948, 842, 729; ^1^H NMR (300 MHz, D_2_O), δ (ppm): 3.96–3.93 (m, ^13^C satellite peaks), 3.75–3.68 (m, 176H, OC*H*_2_C*H*_2_O), 3.65–3.61 (m, 2H, C*H*_2_CH_2_N_3_), 3.53–3.49 (m, 2H, CH_2_N_3_), 3.49–3.46 (m, ^13^C satellite peaks), 3.38 (s, CH_3_O); ^13^C NMR (75 MHz, D_2_O), δ (ppm): 71.7 (CH_3_O*C*H_2_), 70.3 (O*C*H_2_*C*H_2_O), 70.2 (CH_3_OCH_2_*C*H_2_O), 70.0 (O*C*H_2_CH_2_N_3_), 58.8 (CH_3_O), 50.9 (CH_2_N_3_).

### 2.11. Synthesis of Heptakis{2,3-di-*O*-{1′-[methoxypentatetraconta(ethylene glycol)yl-1*H*-1,2,3-triazol-4′-yl]- methyl}}cyclomaltoheptaose (***15***)

A solution of heptakis(2,3-*O*-propargyl)cyclomaltoheptaose **13** (70 mg, 0.042 mmol), 1-azido-1- deoxy-ω-*O*-methoxypentatetraconta(ethylene glycol) **14** (1.439 g, 0.706 mmol), and CuBr (16 mg, 0.118 mmol) in dry DMF (10 mL) under N_2_ atmosphere was stirred at 100 °C for 24 h. After that period of time, starting compound **13** was not detectable by TLC (R_f_ = 0.47 in 30:5:4 EtOAc-EtOH-H_2_O). The reaction mixture was cooled down and the solvent was rotary evaporated. The crude was purified by column chromatography (5:1 MeCN-30% *v*/*v* aqueous NH_3_) to yield compound **15** (716 mg, 0.024 mmol, 57%) as a yellowish powder: mp 51 °C; IR (KBr) ν 3443, 2911, 2878, 1642, 1352, 1102, 952, 580 cm^−1^; ^1^H-RMN (600 MHz, D_2_O), δ (ppm): 8.00 (s, 7H, H-5′), 7.98 (s, 7H, H-5′), 5.25 (bs, 7H, H-1), 4.98 (bs, 7H, OC*H*^a^-C_2_HN_3_), 4.79–4.75 (m, OC*H*^b^-C_2_HN_3_, overlapped with HDO), 4.55 (bs, 14H, CH_2_N), 4.50 (bs, 14H, CH_2_N), 4.05–3.53 (m, H-2,3,4,5,6^a,b^, OC*H*_2_C*H*_2_O), 3.86–3.84 (m, ^13^C satellite peaks), 3.62–3.60 (m, ^13^C satellite peaks), 3.41 (s, 42H, CH_3_O); ^13^C-RMN (150 MHz, D_2_O), δ (ppm): 144.9 (C-4′), 144.3 (C-4′), 125.2 (C-5′), 124.7 (C-5′), 97.6 (C-1), 80.6 (C-3), 78.1–77.8 (C-2,4), 71.0 (*C*H_2_OCH_3_), 69.6–68.7 (C-5, O*C*H_2_*C*H_2_O), 65.9 (O*C*H_2_-C_2_HN_3_), 63.3 (O*C*H_2_-C_2_HN_3_), 60.4 (C-6), 58.1 (CH_3_O), 49.9 (CH_2_N).

### 2.12. Synthesis of Heptakis{2,3-di-*O*-{1′-[methoxypentatetraconta(ethylene glycol)yl-1*H*-1,2,3-triazol-4′-yl]- methyl}}cyclomaltoheptaose Phosphate Sodium Salt (***16***)

P_2_O_5_ (30 mg, 0.208 mmol) was added to dry DMF (2 mL) and the mixture was sonicated in a tightly closed flask for 30 min. Compound **15** (300 mg, 0.010 mmol) was then added to the solution and the resulting mixture was stirred at 40 °C for 3 h. The mixture was cooled down and pH was adjusted to ~7.0 using 1N NaOH. The solvent was rotary evaporated and the residue was dissolved in the minimum amount of H_2_O, syringe filtered (nylon 0.45 μm), and dialyzed (2000 MWCO) against distilled water by changing dialysate solution every 3 h until its conductivity was stable and below 1 μS/cm (5 days for final value of 0.90 μS/cm) to yield **16** (381 mg) as a white solid after lyophilization: mp 49 °C (dec); IR (KBr) ν 3444, 2914, 2879, 1640, 1294, 1255, 1104, 524 cm^−1^; ^1^H-RMN (600 MHz, D_2_O), δ (ppm): 7.92 (bs, 14H, H-5′), 4.79 (bs, H-1, OC*H*_2_-C_2_HN_3_, overlapped with HDO), 4.54 (bs, 14H, CH_2_N), 3.91–3.53 (m, H-2,3,4,5,6^a,b^, OC*H*_2_C*H*_2_O), 3.86–3.84 (m, ^13^C satellite peaks), 3.62–3.60 (m, ^13^C satellite peaks), 3.41 (s, 42H, CH_3_O); ^13^P-RMN (242.9 MHz, D_2_O), δ (ppm): 0.44 (orthophosphates), -9.33 (pyrophosphates), −21.47–(−23.88) (polyphosphates); HR-ICP-MS: P 8.0%.

### 2.13. Synthesis and Characterization of MIL-100(Fe) nanoMOFs

Iron trimesate nanoMOFs were synthesized using a microwave-assisted hydrothermal method described elsewhere [10,15]. Briefly, 30 mL of an aqueous mixture containing 6.0 mM iron chloride hexahydrate and 4.0 mM of BTC was heated at 130 °C under stirring for 6 min by microwave irradiation at 1600 W (Mars-5, CEM Corporation, Matthews, NC, USA). The synthesized nanoMOFs were recovered by centrifugation at 10,000 g for 15 min and purified by six times washing with absolute ethanol. Their morphology was observed with a transmission electron microscope. Mean hydrodynamic diameters and size distributions were determined by DLS. NanoMOFs’ zeta potential (ZP) was measured at 25 °C by DLS in 10 mM PBS pH 7.4. For this, nanoMOFs were diluted to a final concentration of 100 µg/mL using Dulbecco’s PBS solution. The nanoMOF Brunauer–Emmett–Teller (BET) surface area was measured by nitrogen sorption experiments at −196 °C after sample degassing at 100 °C for 15 h under high vacuum. X-ray powder diffraction (XRPD) patterns were recorded for crystallinity characterization. NanoMOFs were stored in ethanol at room temperature, and centrifuged and re-suspended in aqueous media whenever needed.

### 2.14. Surface Modification of MIL-100(Fe) nanoMOFs and Their Characterization

NanoMOFs were centrifuged at 10,000 *g* for 10 min to remove the storage solvent (ethanol) and then redispersed in water by vortex at final concentration of 2 mg/mL. For coating, equal volumes of nanoMOFs suspensions and phosphorylated CD derivatives (PCDs) CD-P, **8**–**10** or **16** solutions at 0.02, 0.2, 0.4, and 0.6 mg/mL (corresponding to nanoMOFs:PCDs 100:1, 10:1, 5:1, and 3:1 mass ratios, respectively) were incubated overnight at room temperature.

Nonattached coating PCDs molecules were removed by centrifugation (10000 g, 10 min). The pellets were dried and the amount of attached PCDs (CD-P, **8**–**10** or **16**) was quantified by ICP-MS. Briefly, uncoated and coated nanoMOFs were digested using aqua regia (15 min under ultrasonic bath), and phosphorous (P) quantification was performed by ICP-MS. Operation conditions were daily optimized using a tuning solution. P isotope was detected using “mass shift mode” (^47^PO^+^) after reaction with oxygen in the cell. Conversely, scandium (Sc) (added as internal standard on samples and calibration standards solutions at a concentration of 10 µg L^−1^) was detected on “mass mode” (^45^Sc^+^). Oxygen was introduced into the collision/reaction cell at a flow rate of 0.35 mL min^−1^. Dwell time for each of the targeted isotopes was 1 s. P was quantified using external calibration prepared using certified 1000 mg L^−1^ P standard solution (Merck, Darmstadt, Germany).

The amount of PCDs associated to nanoMOF was calculated on the basis of their P content by Equation (1):

(1) $$PCDs\;adsorption\;\left(\%\right)\;=\frac{{\left(P\;wt\%\right)}_{sample}}{{\left(P\;wt\%\right)}_{PCDs}}\times 100$$

where (P wt%)_PCDs_ is the phosphorous content in the PCDs coating agents (CD-P, **8**–**10** or **16**).

Uncoated and coated nanoMOFs were characterized to determine their size distribution, morphology, and surface charge. The crystallinity of PCDs-coated nanoMOF was characterized by X-ray powder diffraction (XRPD). The XRPD data were acquired using an in-house setup based on a copper rotating anode generator (RU-200BEH, Rigaku Ltd., Tokyo, Japan). The X-ray beam was filtered and focused by a confocal system consisting of two perpendicular graded multilayer mirrors (CMF-12-38Cu6 from Osmic Inc., Cleveland, OH, USA). The samples were contained in round glass capillaries (WJM-Glas Müller GmbH, Berlin, Germany), 1.5 mm in diameter, and mounted on a homemade motorized sample holder. The scattering signal was detected by a CCD detector cooled down to −30 °C (Photonic Science, Saint Leonards-on-sea, UK). The sample-to-detector distance was 296 mm, yielding an accessible scattering vector range of 0.035–0.5 Å^−1^. The images were integrated, corrected, and calibrated using the Nika suite (version 1.74) running in Igor Pro 7.08.

The PCDs-coated nanoMOF colloidal stabilities were estimated in complete cell culture medium by measuring hydrodynamic diameters using DLS after 6 h incubation at 37 °C in cell culture media.

### 2.15. Quantification of nanoMOFs Uptake by Macrophage Cells

Macrophage cells (J774A.1) were seeded in 24-well plates at a density of 3.0 × 10^5^ cells per well in cell culture medium and kept overnight at 37 °C in 5% CO_2_. Cells were then incubated with 1 mL cell culture media containing uncoated or coated nanoMOFs (50 µg mL^−1^) for 4 h. After incubation, the cells were washed with PBS three times to eliminate the nanoMOFs which did not interact with the cells. Cells were finally dried and digested using aqua regia (15 min under ultrasonic bath). Fe quantification was performed by ICP-MS. Operation conditions were daily optimized using a tuning solution. Fe and Co isotopes (added as internal standard on samples and calibration standards solution at a concentration of 10 µg L^−1^) were detected using “on-mass mode” (^54^Fe^+^, ^56^Fe^+^, ^59^Co^+^). Helium was introduced into the collision/reaction cell at a flow rate of 3 mL min^−1^. Dwell time for each of the targeted isotopes was 1 s. Fe was quantified using external calibration prepared using certified 1000 mg L^−1^ Fe standard solution (Merck, Darmstadt, Germany).

### 2.16. Surface Modification of Doxorubicin (DOX)-Loaded MIL-100(Fe) Nanomofs with ***9***

An ethanolic suspension of MIL-100 (Fe) nanoMOFs was centrifuged (10 min, 10,000 *g*) and the sediment redispersed in MilliQ water. The nanoMOF aqueous suspension was further mixed with a DOX aqueous solution in 1:5 DOX:nanoMOFs weight ratio. The mixture was maintained under gentle stirring for 48 h at room temperature. The DOX-loaded nanoMOFs were recovered by centrifugation (10 min, 10,000 *g*) and the supernatants were used to quantify the drug payload according to Equation (2):

(2) $$Payload\;\left(\%\right)=\;\frac{Encapsulated\;Drug\left(mg\right)}{nanoMOFs\;\left(mg\right)}\;\times 100$$

The DOX-loaded nanoMOFs were further surface modified by incubation with an aqueous solution of **9** overnight. After surface functionalization, DOX-loaded nanoMOFs@**9** were recovered by centrifugation (10 min, 10000 g). The supernatant was also recovered to quantify the DOX amount.

### 2.17. Isothermal Titration Calorimetry (ITC) Measurements

*Canavalia ensiformis* (Jack bean) concanavalin A (ConA) lectin (Sigma, type VI, lyophilized powder) was used as received. All solutions were prepared in buffered MilliQ water (18.2 MΩcm), degassed for 10 min, and thermostated at 25 °C prior to each experiment. In the case of conjugates **8** and **10**, 20 mM phosphate buffer (pH 7.2) was used for both lectin and conjugates, which were considered as analyte and titrating agents, respectively. On the other hand, 10 mM TRIS buffer (pH 7.5) was employed for both lectin and nanoMOFs@**8** and nanoMOFs@**10**, which were defined as titrating agent and analytes, respectively. In all cases, lectin solution concentrations were determined by UV-Vis spectroscopy (A^1%^_280 nm_ = 13.7 for the tetrameric form). ITC experiments were conducted as previously described elsewhere [50]. After calibrating the calorimeter as recommended by the manufacturer, the reference and sample cells were filled with the corresponding pure buffer and the analyte solution, respectively, while a 250 μM syringe was filled with the solution of the respective titrating agent (Supplementary Materials, Table S2). The sample cell solution was continuously stirred at 416 rpm while the titrating agent solution was injected in 8 μL aliquots every 5 min. The titrating agents were also injected into the corresponding buffer in separate experiments in order to obtain the dilution background profiles, which turned out to be similar to the heat signals detected after saturation in the interaction experiments with the analytes. Obtained thermograms depicted the transfer of heat per second following each injection of the titrating agent into the analyte solution as a function of time. The integration of each peak gave the amount of heat generated by each injection after subtracting the titrating agent dilution heat. The best fit of the experimental data to the model of *n* equal and independent sites provided the binding constant and the thermodynamic profile along with the corresponding standard deviations. For these calculations, we assumed that Δ*H* = Δ*H*^0^, while the changes in the standard free energy (Δ*G*^0^) and entropy (Δ*S*^0^) were calculated as Δ*G*^0^ = −R*T*ln*K* and *T*Δ*S*^0^ = Δ*H* − Δ*G*^0^, respectively.

## 3. Results and Discussion

### 3.1. Synthesis of Phosphate β-CD Derivatives (PCDs)

**Scheme 1:**
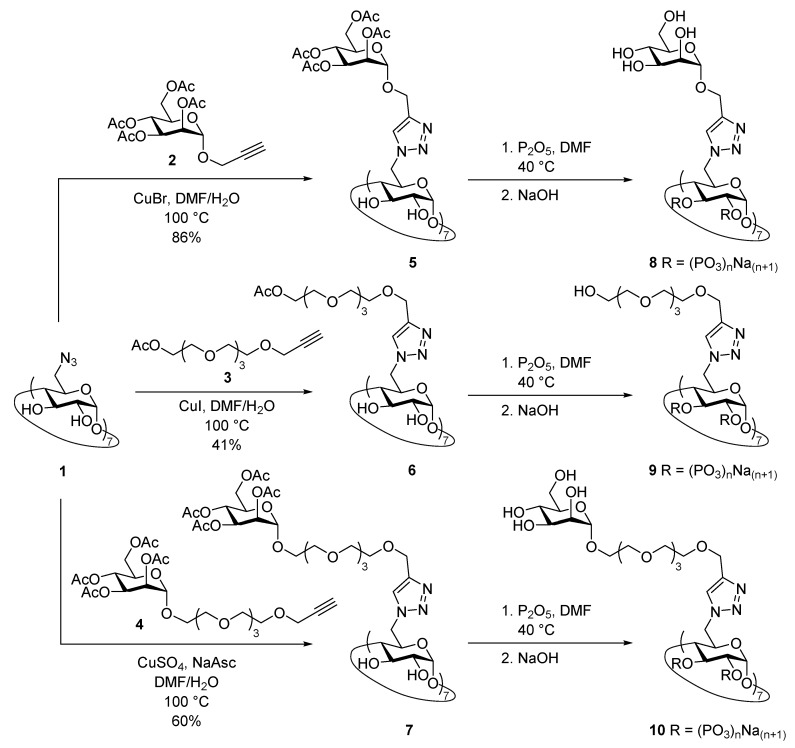
Synthesis of per-functionalized β-cyclodextrin (β-CD) phosphate derivatives **8**–**10**.

We carried out the synthesis of per-functionalized β-CD phosphate derivatives from per-6-azido-6-deoxy-β-CD **1** (Scheme 1) for their use as coating agents in the postsynthetic surface modification of the iron-based nanoMOFs MIL-100(Fe). Per-6-azido compound **1** was a convenient starting material for the attachment on its primary face of seven appendages by Cu(I)-catalyzed alkyne-azide cycloaddition reaction (CuAAC) [51,52,53]. As its azide counterpart, a terminal alkyne group was required on the motifs intended to be attached to the macrocycle. Thus, we performed the reaction of **1** with acetylated propargyl derivatives **2**–**4** containing mannose and/or TEG residues.

We first tested the synthesis of compound **5** with seven acetylated mannopyranose residues by coupling propargyl 2,3,4,6-tetra-*O*-acetyl-α-d-mannopyranoside (**2**) to perazide **1** in the presence of CuI(C_2_H_5_O)_3_P as catalyst in DMF at 100 °C. These conditions afforded a mixture, whose TLC showed different spots with very close R_f_ which turned out to be very difficult to separate by column chromatography using 25:1:5 CH_3_CN-H_2_O-(30% *v*/*v* aq. NH_3_) as eluent. We observed that small variations in the eluent proportions sharply changed the column performance. However, a small amount of the pure product was obtained, and HPLC-MS and MALDI-TOF mass spectra allowed the detection of the target compound together with a number of under-substituted analogues, including one derivative bearing an amino group as a result of a partial reduction. In order to optimize the conversion to **5**, other copper catalysts (CuI, CuBr), solvents, and reaction temperatures were tested. By performing the reaction with CuBr in a 3:7 H_2_O-DMF mixture, the desired compound was obtained as a white solid in 86% yield after a purification that included removal of copper traces. The molecular weight of **5** was confirmed by MALDI-TOF mass spectrometry, which showed the *m*/*z* pick corresponding to [M+Na]^+^ ion. The formation of the triazole rings was confirmed by ^1^H NMR spectroscopy as it showed a peak at δ 7.96 ppm corresponding to the triazole proton along with the absence of the triplet at δ 2.47 ppm of the alkyne proton of the starting mannoside. In addition, ^13^C NMR spectrum also showed peaks at δ 141.9 and 125.7 ppm corresponding to the C-4’ and C-5’ of the triazole rings, respectively. Both ^1^H and ^13^C NMR spectra also revealed DMF residual peaks (Supplementary Materials, Figures S2 and S12). The efforts made to remove the solvent from the sample, including lyophilization, azeotropic distillation with toluene, and high vacuum at 50 °C, turned out to be unsuccessful. It is noteworthy that NMR spectra for **5** were recorded in DMSO-*d*_6_ at 80 °C to overcome the conformational energy barriers of the molecule. NMR spectra at 25 °C showed several sets of signals indicating that the interconversion among different conformers of the structure were slow enough in the time scale of the experiments, probably due to the steric hindrance on the primary face of the macrocycle. In sharp contrast, increasing the temperature to 80 °C led to well-defined signals for each nuclei, confirming the *C*_7_ symmetry of the molecule, and hence the presence of seven appendages on the macrocycle.

The synthesis of compound **6** bearing seven acetylated tetraethylene glycol branches also turned out to be troublesome. A set of catalysts and conditions were tested, and the obtained crude products were analyzed by TLC and HPLC-MS. Among the screened sets-up, CuI in 1:1 DMF-H_2_O at 100 °C was found to be optimal for the cycloaddition reaction, affording **6** in 41% yield after purification. The low yield can be attributed to a partial deacetylation during the column elution, judging from TLC (5:1 1,4-dioxane-30% *v*/*v* aq. NH_3_). MALDI-TOF confirmed the molecular weight of the target compound, which showed the *m*/*z* peak corresponding to [M + Na]^+^ ion. The formation of the triazole rings (peak at δ 7.86 ppm in the ^1^H-NMR spectrum) and the presence of acetyl moieties (peaks at δ 2.00 ppm in ^1^H NMR spectrum, and at δ 169.6 (carboxyl) and 20.0 ppm (methyl) in ^13^C NMR spectrum) were unambiguously confirmed by NMR spectroscopy. As in the case of derivative **5**, recording the NMR spectra in DMSO-*d*_6_ at 80 °C was mandatory in order to obtain single, well-defined signals for nuclei of the glucose units of the macrocycle, thus confirming the *C*_7_ symmetry of the molecule due to the per-modification at C-6 positions.

Next, CD derivative **7** (Scheme 1) decorated with seven acetylated mannose residues linked through a tetraethylene glycol (TEG) tether was prepared. In this case, among the tested reaction conditions the use of CuSO_4_ and sodium ascorbate as catalyst promoters in 2:1 DMF-H_2_O at 100 °C resulted to be optimal and gave **7** in 60% yield after purification. The structure of **7** was confirmed by MALDI-TOF mass spectrometry and NMR spectroscopy. ^1^H NMR spectrum of **7** measured in DMSO-*d*_6_ at 80 °C showed a singlet corresponding to the triazole hydrogen at δ 7.86 ppm, two doublets at δ 5.09 and 4.90 ppm corresponding to the anomeric protons of the glucose units of the macrocycle (H-1) and the mannose residues (H-1‴), respectively, and four singlets at δ 2.10–1.93 ppm assignable to the 28 acetyl groups of the mannose units. The presence of the TEG tether was confirmed by the multiplet that appeared in the range of δ 3.57–3.48 ppm corresponding to the CH_2_ groups of chain.

β-CD derivatives **5**–**7** presented fourteen free OH groups on the secondary face of the macrocycle still available for further modifications. In order to transform these compounds into potential coating agents for iron-based nanoMOFs MIL-100(Fe) [15,17], we decided to phosphorylate those OH to yield phosphates **8**–**10** (Scheme 1). Phosphorylations of **5**–**7** were carried out by treatment with an excess of P_2_O_5_ in DMF at 40 °C. After the starting material was consumed, pH was increased to ~13 to remove the acetyl groups of the primary face appendages. The crude products were dialyzed towards distilled water. Based on the theoretical molecular weight of phosphates **8**–**10** by considering fourteen disodium phosphate groups on the secondary face (4572.71–5804.73 Da), membranes of 500–1000 Da MWCO were initially chosen. However, intense peaks of unknown impurities at around δ 8.40, 3.30, and 2.70 ppm were observed in the ^1^H NMR spectra of the dialyzed products. Increasing membrane pore size to 2000 MWCO effectively removed the peaks at around δ 8.40 and 3.30 ppm, although it did not affect to the signal at ca. δ 2.70 ppm and significantly decreased the obtained amount of compounds **8**–**10**. Both ^1^H and ^13^C NMR spectra of **8**–**10** confirmed the absence of the peaks corresponding to acetyl groups presents in the starting materials **5**–**7** and showed broad signals for the nuclei of both the macrocycle and the appendages. It is important to highlight that random phosphorylation with P_2_O_5_ yields a mixture of ortho-, pyro-, and polyphosphates depending on the molar ratio between the hydroxyl groups and the added amount of P_2_O_5_, but also on the presence of traces of water during the reaction. Indeed, ^31^P NMR spectra showed, in all cases, three sets of signals at around δ 0.5, −8.0, and −22.5 ppm corresponding to the ortho-, pyro-, and polyphosphate species, respectively [54], formed on the secondary face of the macrocycles. Furthermore, we determined the contents of P in derivatives **8**–**10** by HR-ICP-MS and used those data to calculate the number of phosphate groups (Supplementary Materials, Table S1). Assuming that all OH groups of the secondary face were phosphorylated, an average number of phosphate groups per OH of 2.55, 2.43, and 5.83 for compounds **8**, **9**, and **10**, respectively, was found, indicating that all ortho-, pyro-, and polyphosphate species were most probably present as ^31^P NMR spectra previously suggested.

**Scheme 2:**
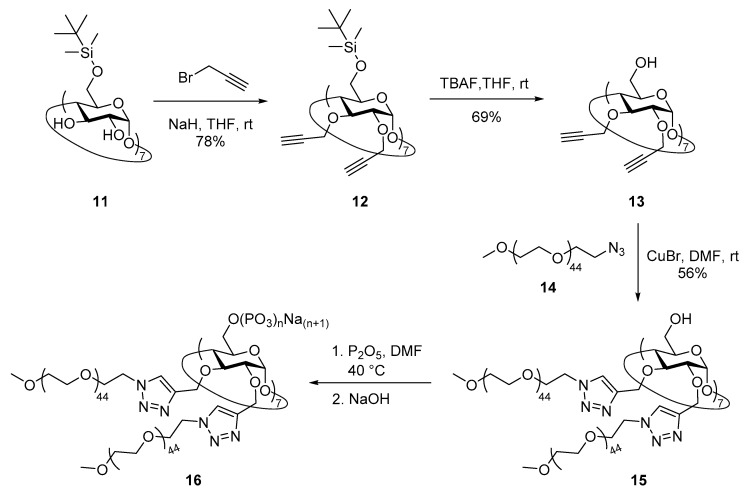
Synthesis of per-functionalized β-CD phosphate derivative **16**.

In a similar strategy, per(6-*O*-*tert*-butyldimethylsilyl)-β-CD **11** was used as starting material for the attachment of methoxy poly(ethylene glycol) branches on the secondary rim of the macrocycle and subsequent phosphorylation of the primary face (Scheme 2). Compound **11** was prepared by reacting β-CD with TBDMSCl in pyridine as previously reported [49], although a new rapid and reproducible purification method was developed. Thus, enough TBDMSCl was added in portions until TLC (30:5:4 EtOAc–96% *v*/*v* EtOH–H_2_O) showed no undersilylated species (see Experimental part). Purification of **11** was easily performed by a short flash chromatography using first the quaternary mixture of solvents 40:40:20:4 CH_2_Cl_2_–MeCN–96% *v*/*v* EtOH–30% *v*/*v* aqueous NH_3_ to rapidly elute the oversilylated byproducts, and then using 40:40:20:4 CH_2_Cl_2_–MeCN–96% *v*/*v* EtOH–H_2_O to quickly wash out highly pure silyl **11** in 86% yield. Extensive propargylation and subsequent desilylation of cyclooligosaccharide **11** according to published procedures [47] led to derivative **13**, which in turn was reacted with 1-azido-1-deoxy-ω-*O*-methoxypentatetraconta(ethylene glycol) **14** (average MW ~2000) in the presence of CuBr as catalyst in DMF at 100 °C to give compound **15** in 57% yield after column chromatography. ^1^H NMR spectrum of compound **15** showed two sharp peaks at δ 8.00 and 7.98 ppm for the protons of the triazole rings located at C-2 and C-3 positions of the glucose residues of the macrocycle, suggesting a *C*_7_ symmetry and, hence, the cycloaddition of fourteen PEG chains on the secondary face of the β-CD. However, broad signals were found for the skeletal protons of the glucose moieties, which can be reasoned in terms of macrocycle rigidity due to the high steric hindrance of the molecule. The same effect was visible in the ^13^C NMR spectrum, with well-defined signals for triazole rings but broad peaks for the glucose residues nuclei. Interestingly, the intense signals arising from methylene groups of the fourteen long PEG chains dominated the dynamic range in both ^1^H and ^13^C spectra. As a result, ^13^C satellite peaks could be observed as multiplets at δ 3.86–3.84 and 3.62–3.60 ppm in ^1^H NMR [55], as well as a set of sideband peaks symmetrically located at both sides of the PEG signal (δ 69.6 ppm) at chemical shifts that were whole multiples of ~315 Hz in ^13^C NMR.

Phosphorylation of the OH groups of the primary face of **15** was achieved by treatment with P_2_O_5_ in DMF at 40 °C as described above, although in this case pH was directly adjusted to ~7.0 after reaction as no acetyl groups were present on the starting material (Scheme 2). Dialysis towards distilled water through 2000 MWCO membrane finally achieved phosphate **16** after lyophilization. ^1^H NMR spectrum showed less defined signals for both cyclooligosaccharide skeleton and triazole rings than that for the starting material **15**, evidencing an increase of the structure rigidity. Once again, the signal from PEG methylenes was very intense and accompanied by ^13^C satellite peaks as multiplets at δ 3.86–3.84 and 3.62–3.60 ppm. Furthermore, the effect of the signal arising from those groups on the dynamic range of the ^13^C NMR spectrum was so strong that it was not possible to see the rest of the molecule peaks even after long acquisition periods. ^31^P NMR spectrum showed very intense signals at around δ −22.5 ppm along with much smaller peaks at δ 0.44 and −9.33 ppm peaks, suggesting that phosphorylation of primary face afforded mainly polyphosphate groups together with a few ortho- and pyrophosphate functionalities [54]. This result was also confirmed by HR-ICP-MS data for the phosphorus contents of derivative **16**, which allowed us to calculate an average number of 15.21 phosphate residues per primary OH group. This number is in sharp contrast with those obtained for derivatives **8**–**10** varying between 2.43 and 5.83 despite the fact that a similar molar ratio of P_2_O_5_ per free OH (between 2.3 and 4.4) was employed in the four cases.

### 3.2. MIL-100(Fe) nanoMOFs Synthesis and Surface Modification

**Figure 1:**
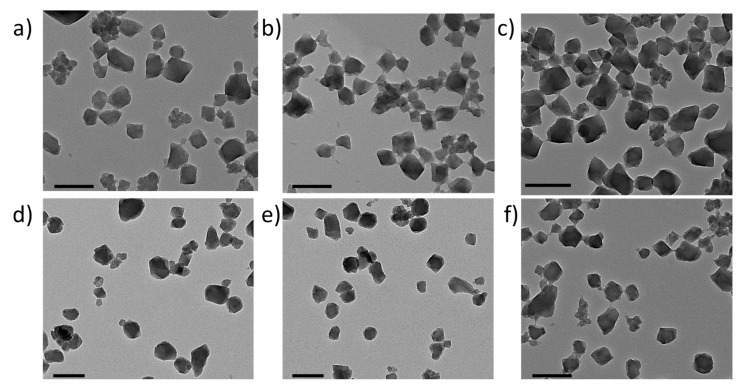
TEM images of nanosized metal-organic frameworks (nanoMOFs) before and after coating step: (**a**) Uncoated nanoMOFs; (**b**) nanoMOFs@CD-P; (**c**) nanoMOFs@**8**; (**d**) nanoMOFs@**9**; (**e**) nanoMOFs@**10**; (**f**) nanoMOFs@**16**. Scale bar: 200 nm.

MIL-100(Fe) nanoMOFs with a mean hydrodynamic diameter of 231 ± 14 nm and BET surface area of 1690 ± 80 m^2^ g^−1^ were successfully synthesized by an organic solvent-free microwave-assisted hydrothermal method [15]. Resulting nanoMOFs had typical facetted structures (Figure 1a) and showed XRDP similar to those previously published (Supplementary Materials, Figure S24). Surface modification of MIL-100(Fe) nanoMOFs with PCDs was carried out through a “green” (meaning organic solvent-free) method consisting of impregnation of nanoMOFs in aqueous solutions of PCDs CD-P, **8**–**10** or **16** at different mass ratios nanoMOFs:PCDs, ranging from 100:1 to 3:1, to yield nanoMOFs@PCD core-shell. Both uncoated and coated MIL-100(Fe) nanoMOFs were characterized by a set of complementary techniques. Even at the 3:1 nanoMOFs:PCDs mass ratio the particles maintained their facetted structures (Figure 1b–f). No morphological differences were found between coated and uncoated particles, independently of the coating material. Indeed, XRDP studies indicated that the crystalline structure of MIL-100(Fe) nanoMOFs was preserved after surface modification (Supplementary Materials, Figure S24) in spite of the relatively high amounts of PCDs associated to the nanoMOFs (see below). For instance, the strong reflections present in the uncoated sample were preserved in the treated ones, showing that the integrity of the lattice was preserved in the latter, which did however exhibit a supplementary flat background at wide angles and a stronger power-law contribution at small angles, consistent with the presence of an additional amorphous component. These observations are in agreement with previously reported data on different structures used as coating for the same nanoMOFs [15,17]. The hydrodynamic diameters of MIL-100(Fe) nanoMOFs in water were determined by DLS before and after surface modification. Measurements showed neither mean size nor polydispersity significant variations after the coating process, independently of the concentration and type of coating material. Mean diameters increased from 224 ± 21 nm for uncoated nanoMOFs to 229 ± 18, 248 ± 25, 239 ± 17, 237 ± 19, and 241 ± 23 for nanoMOFs coated with CD-P, **8**–**10** and **16**, respectively. These data indicated that the coating thickness was less than 15 nm in all cases, and that no aggregation occurred. Similar behavior was found in DMEM biological medium complemented with 10% FBS (Supplementary Materials, Figure S25). Again, no trace of aggregation was observed as the mean diameters remained constant after 6 h incubation at 37 °C. These results allow for biological studies on the interactions of coated nanoMOFs with macrophages.

**Figure 2:**
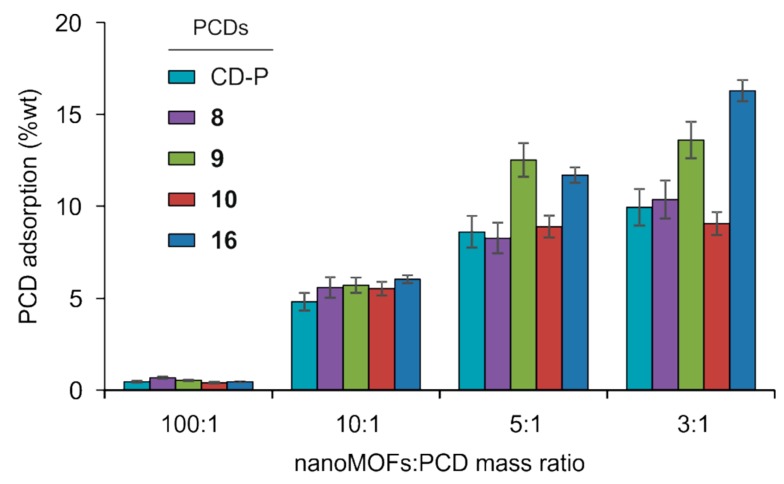
Phosphate β-CD derivatives (PCDs) grafting (wt%) versus nanoMOFs:PCDs mass ratio assayed.

The amount of PCDs associated with the nanoMOFs was determined by direct quantification of the P content in the coated samples by ICP-MS after sample digestion, taking advantage of the fact that P is the only element present in the PCDs derivatives but not in the nanoMOFs. As it can be seen in Figure 2, the amount of PCDs attached to nanoMOFs was directly proportional to the amount of the coating material employed for the nanoMOFs coating procedure. In parallel, the grafting efficiencies of the various PCDs derivatives decreased from ~70 to ~35% with increasing the amount of PCDs in the preparation procedure (Supplementary Materials, Figure S26). In the case of CD-P and **8**–**10**, the grafted amounts tended to reach a plateau when a 3:1 nanoMOFs:PCDs mass ratio was used. Thus, CD-P and derivative **8** decorated with seven mannose residues directly attached to its primary face reached a value of 10 ± 1 wt% of grafting, while PCD **10** coated the nanoMOFs in less extension (9 ± 1 wt%). In contrast, PCD **9** decorated with seven flexible and less bulky TEG chains was adsorbed onto the nanoMOF surface at 14 ± 1 wt%, suggesting that steric hindrance of the coating structure might correlate with the nanoMOFs covering density. However, PCD **16** having fourteen long PEG chains on the secondary face showed the highest covering value of the series (16 ± 1 wt% at a 3:1 nanoMOF:**16** mass ratio). Nevertheless, the amounts of coating PCDs were in the same range of previously reported values for other PCDs derivatives on the same MIL-100(Fe) nanoMOFs (~17 wt%) [17].

**Figure 3:**
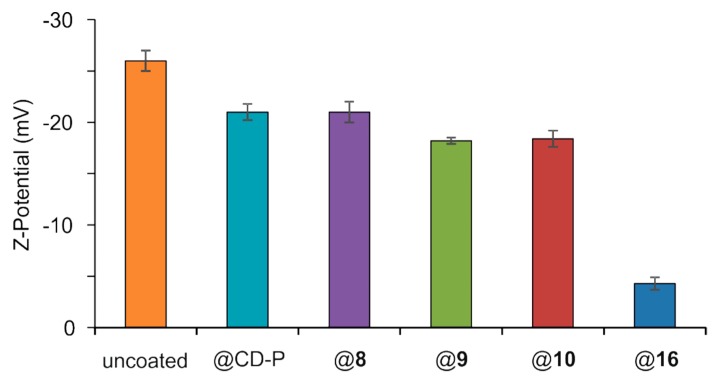
Zeta potential of nanoMOFs coated or not with PCDs in 10 mM PBS at pH 7.4.

Lack of colloidal stability is an important drawback hampering nanoparticle biomedical applications. Zeta potential analysis gives values of the nanoparticles global charge, which depends on pH, presence of ions, and nanoMOFs concentration. Measurements were performed at pH 7.4 and reflect the stability of the system. Results are depicted in Figure 3, which shows that the ZP values shifted from −26 ± 1 mV for the uncoated nanoMOFs to values between −21 ± 1 and −18 ± 1 mV for the nanoMOFs covered with PCDs CD-P and **8**–**10**. However, when PCD **16** was used, coated nanoMOFs reached ZP values close to 0 (−4 ± 1 mV). This behavior is a clear indication that the carboxylate surface groups on the nanoMOFs were shielded by the nonionic PEG chains. These observations are in agreement with previously reported ZP values close to zero in the case of other types of PEG-coated nanoparticles [37].

### 3.3. ITC Experiments on ConA Biorecognition

Mannose-coated MIL-100(Fe) nanoMOFs are the result of the self-assembly of multiple heptavalent mannose clusters (**8** and **10**) on the surface of the nanoparticles. Displaying a multivalent presentation of mannoside ligands would be equivalent to an artificial “glycocalix” [40]. To test the molecular recognition properties of such mannose-coated nanoMOFs, we studied the binding affinity of glycoclusters **8** and **10**, as well as mannose-coated nanoMOFs@**8** and nanoMOFs@**10**, towards the model mannose-specific lectin concanavalin A (ConA) by using isothermal titration calorimetry (ITC). This technique allows the quantification of the stoichiometry, affinity, and thermodynamic profile for the interaction between ligands and receptors by detecting the heat released or absorbed during the binding event. Indeed, ITC directly provides values for the enthalpy change of binding (Δ*H*), while the binding constant (*K*) and the stoichiometry (*n*), defined as the ratio between the concentration of the conjugates and the protein when the lectin binding sites are saturated, are estimated from the best fit to a model of *n* equal and independent sites by a nonlinear least square algorithm. It is important to highlight that the selected model lectin, ConA, and the actual human macrophage mannose receptor (hMMR) are different, both at structural and binding mechanism levels, and thus, any extrapolation from the former to the latter should be made with care. However, recently it has been reported that affinities of a series of mimics for both lectins in solution are qualitatively similar [56,57], which supports our choice.

**Figure 4:**
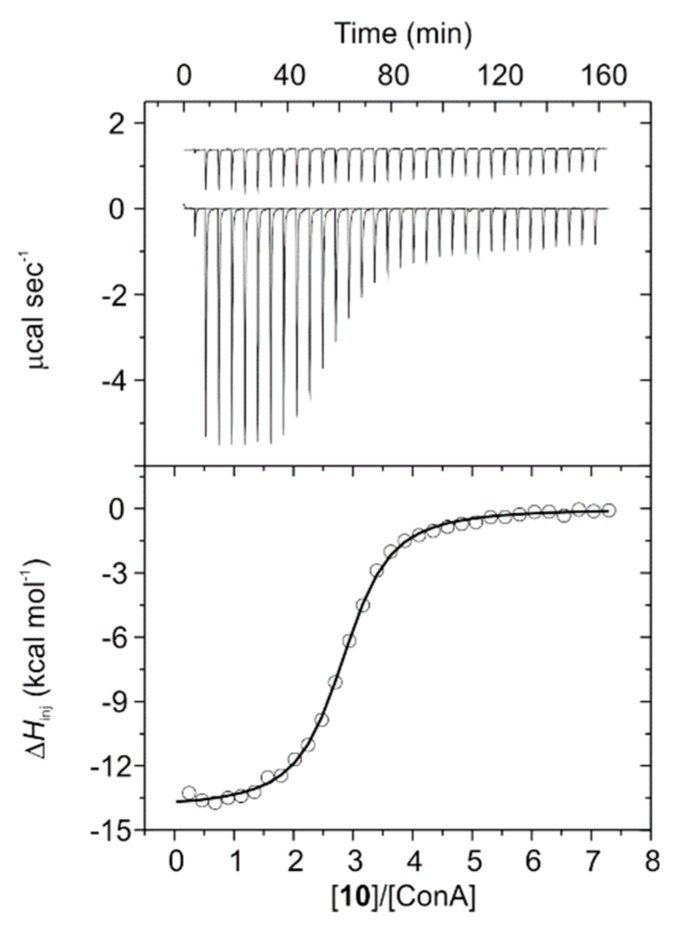
Titration of concanavalin A (ConA) with conjugate **10** in 20 mM phosphate buffer (pH 7.2) at 25 °C. The top panel shows the raw calorimetric data denoting the amount of generated (negative exothermic peaks) heat following each injection of the conjugate. The area under each peak represents the amount of heat released upon binding of the conjugate to the lectin. Note that as the titration progresses, the area under the peaks gradually becomes smaller because of the increasing saturation of the sugar binding sites of the protein. This area was integrated and plotted against the molar ratio of the conjugate to ConA (as tetramer). The smooth solid line represents the best fit of the experimental data to the model of *n* equal and independent binding sites.

**Figure 5:**
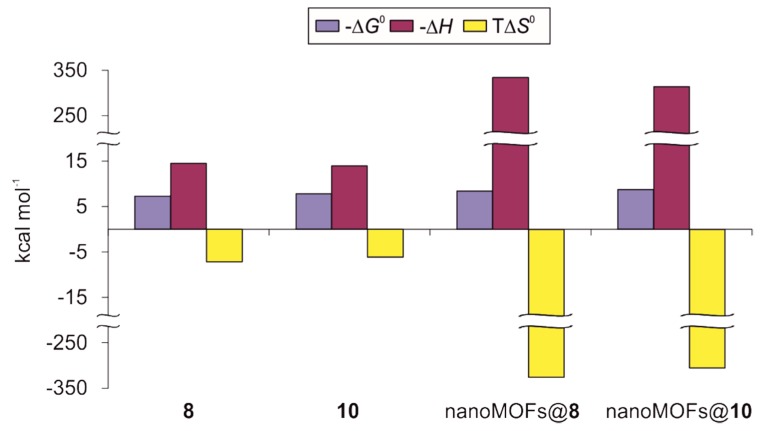
Thermodynamic profiles comprising free energy (−Δ*G*^0^), enthalpy (−Δ*H*), and entropy changes (*T*Δ*S*^0^) for the interaction of conjugates **8**, **10**, nanoMOFs@**8**, and nanoMOFs@**10** with ConA in 20 mM phosphate buffer (pH 7.2) at 25 °C for **8** and **10**, and 10 mM TRIS buffer (pH 7.5) at 25 °C for nanoMOFs@**8** and nanoMOFs@**10**.

Titration experiments for mannosylated PCDs **8** and **10** were performed in 20 mM phosphate buffer at pH 7.2 at 25 °C. Aliquots of each PCD were injected into a solution of ConA within the calorimeter cell and the amount of released heat following each injection was plotted against the molar ratio between the conjugate and the lectin (Figure 4 and Figure S27). In addition, control experiments were performed by injecting ligands into the cell containing buffer without lectin and showed insignificant heats of dilution. Thermodynamic profiles calculated by fitting the obtained data to the model can be found in Table 1 and Figure 5, and are in sharp agreement to those typically reported for protein-carbohydrate binding [50,58]. Indeed, ITC experiments showed clearly exothermic interactions with ConA for both conjugates **8** and **10**. Thus, their thermograms were characterized by negative peaks corresponding to a release of heat associated with the binding interaction, which diminished progressively due to the gradual saturation of the protein binding sites. Thermogram for PCD **10** reached a plateau at lesser concentration ratio than that for **8**, which can be reasoned taking into account that the binding constant for the former is 2.3-fold higher than that for the latter (Table 1). Both thermodynamic profiles were very similar and enthalpy-driven (Figure 5), comprising favorable (negative) enthalpic terms partially counterbalanced by smaller unfavorable (negative) entropic contributions.

Interestingly, binding enthalpy for conjugate **8** was slightly more favorable (~4%) than that for **10**, which may suggest a better docking of the mannose moieties of the former to the carbohydrate-binding sites of the protein. However, entropic unfavorable term was ~17% larger in the case of **8**, resulting in a compensation of ~50% of the enthalpy contribution for this conjugate while **10** showed an entropic counterbalance of only ~44%. This fact explains why the interaction was stronger in the case of conjugate **10** as their respective binding constants reveal, and it was clearly related to the presence of tetra(ethylene glycol) spacers between the mannose residues and the cyclooligosaccharide in its structure. These tethers most probably increased the distance and mobility of the monosaccharides around the primary face of the macrocycle, moderating the rigidity gain after protein binding and thus softening the concomitant entropic lost. Previous reports have shown the beneficial effects of introducing flexible and relatively long bridges between a β-CD core and sugar ligands on their ability to bind lectins in solution [59,60]. Regarding the stoichiometry of the interactions, the ratios between the concentration of the conjugates and the protein when the system reaches saturation (*n*) were calculated as 2.44 and 2.79 for compounds **8** and **10**, respectively (Table 1). ConA is well-known to present a tetrameric structure at pH values above 7.0, with a unique mannose-binding site per monomer [61]. Thus, *n* values below 4 suggest that not all binding sites of the protein were occupied when the system reached saturation, which might be reasoned in terms of crowded spatial arrangements taking into account the sharp differences in sizes between the lectin and the mannose-containing phosphates **8** and **10**.

In contrast to **8** and **10**, ITC experiments with nanoMOFs@**8** and nanoMOFs@**10** were performed in 10 mM TRIS (pH 7.5) buffer due to the fact that phosphate buffer used above could compete for the iron sites within the nanoparticles surface, causing the coating depletion. Furthermore, inverse titration was required for nanoMOFs@**8** and nanoMOFs@**10** since colloidal stability for these nanoparticles had only been tested at concentrations below 1 mg/mL (~21 and ~15 μM of coating PCDs **8** and **10**, respectively, on the nanoMOFs surface) which turned out to be too low when direct titrations were attempted. Thus, in these cases aliquots of solutions of ConA lectin in concentration below its aggregation limit (80 μM as tetramer, in our hands) were injected within nanoparticle colloidal suspensions contained in the calorimeter cell. To the best of our knowledge, this was the first time that the interaction of nanoMOFs coated with biorecognizable moieties towards their biological receptor counterpart was evaluated by this technique. As previously discussed for free PCDs **8** and **10**, obtained thermograms for nanoMOFs@**8** and nanoMOFs@**10** were strongly exothermic, presenting negative peaks that become shorter after each injection (Supplementary Materials, Figure S27). Again, fitting obtained data to the model of *n* equal and independent binding sites gave us enthalpy-driven thermodynamic profiles for both coated systems (Table 1 and Figure 5). Noticeably, binding enthalpies turned out to be ~23-fold more favorable than those previously measured for the free coating agents **8** and **10**, reaching values more negative than −300 kcal mol^−1^. Similarly, unfavorable entropic terms were of the same order of magnitude, compensating ~97% of the enthalpic contributions in both cases. Such behavior has also been reported in the case of gold nanoparticles coated with sugars when interacting with ConA [62], and it was most likely a consequence of the high density of ligands present on the nanoparticle surface resulting in a remarkable multivalent effect. In absolute terms, calculated Δ*G*^0^ values were 12–15% more favorable for the nanoparticles than those for the free phosphates, and thus the binding constants for nanoMOFs@**8** and nanoMOFs@**10** were 6.33-fold and 4.8-fold larger, respectively. Once more, the binding affinity of nanoMOFs@**10** presenting TEG spacers was ~2-fold stronger than that for nanoMOFs@**8**, where the mannose residues were directly attached to the primary face of the macrocycle, most probably explained through the better fit of the ligand associated with the higher flexibility of the linker, reducing the steric hindrance. Again, *n* values for nanoMOFs@**8** and nanoMOFs@**10** were below 4 when interacting with the lectin (0.90 and 1.06, respectively), suggesting an extensive covering of the coated nanoparticles surface by protein tetramers at the system saturation point. This was possible due to the fact that the nanoparticles showed an average diameter of 231 nm, which was ~29-fold larger than the lectin, having a reported hydrodynamic diameter of ~8 nm [63].

Finally, it should be underlined that despite the sharp differences in the values of the thermodynamic parameters calculated for **8**, **10**, nanoMOFs@**8**, and nanoMOFs@**10**, all evaluated mannoside derivatives showed typical energetic profiles for protein-carbohydrate association events [50,58]. Indeed, the enthalpy–entropy compensation was strongly linear with a slope of ~1.00 and a correlation coefficient of 1 (Supplementary Materials, Figure S28).

### 3.4. Interactions of Surface-Modified nanoMOFs with a Macrophage Cell Line

**Figure 6:**
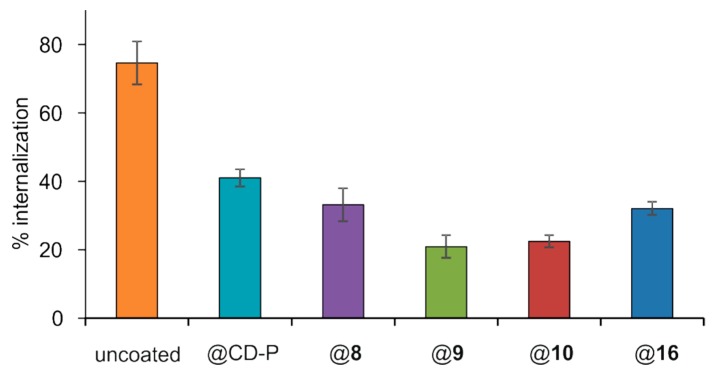
In vitro interaction of uncoated and coated nanoMOFs with PCDs CD-P, **8**–**10**, and **16** with a J774A.1 macrophage cell line. 50 µg/mL nanoMOFs were incubated with 3 × 10^5^ J774A.1 cells for 4 h, and then washed to remove the nonfirmly bound nanoMOFs. After cell lysis, the amount of internalized nanoMOFs was determined by ICP-MS and was expressed as a % of the initial amount put in contact with the cells.

Once demonstrated by ITC that mannose-coated nanoMOFs recognized a specific protein receptor with enhanced avidity, we evaluated the interactions of nanoMOFs coated with **8**–**10** and **16** with macrophages on the murine macrophage cell line J774A.1. To compare the efficacy of different coatings, an incubation time of 4 h was chosen. Indeed, for uncoated MIL-100(Fe) nanoMOFs, this time corresponded to an efficient uptake by J774A.1 macrophages [64] as is in the order of magnitude of blood circulation times of PEG-coated nanoparticles [20]. Quantitative measurement on the amounts of nanoMOFs uptaken in cells were obtained by ICP-MS, after contact of nanoparticles with cells followed by extensive washing to remove the nonassociated particles. As shown in Figure 6, around 75% of uncoated nanoMOFs were taken up in macrophages after 4 h incubation, corresponding to around 23 µg nanoMOFs/3 × 10^5^ cells. This result demonstrates that macrophages avidly take up nanoMOFs, in agreement with reported data [64]. Remarkably, the presence of PCDs **8**–**10** or **16** on the nanoparticles surface significantly reduced the nanoMOFs internalization. The nanoMOFs amounts uptaken by the cells decreased in the order CD-P > **8** > **16** > **10** > **9**, with uptake percentages of 41 ± 3, 33 ± 5, 32 ± 2, 22 ± 2, and 21 ± 3%, respectively (Figure 6). These results indicated that all of the PCDs coatings played a role on the nanoMOFs cell internalization, although the relationship between the uptaking reduction and the structure is not obvious.

Interestingly, an almost two-fold reduction of internalization was observed for the nanoMOFs coated with CD-P without any further modification (from 75% for uncoated nanoMOFs to 41% for nanoMOFs coated with CD-P). This was a surprising result as to the best of our knowledge no study has reported yet that a CD coating could reduce macrophage uptake. When CD was further functionalized with PEG chains (**16**), which are well known to reduce macrophage uptake [65], a better “stealth” effect was obtained with around 32% cell association compared to 41% for nanoMOFs coated with CD-P. A similar effect was obtained in the case of PCD **8** (~33%), where mannose was attached to the macrocycle instead of PEG chains. The most efficient “stealth” behavior was observed for nanoMOFs@**9** and mannose-grafted nanoMOFs@**10**, both characterized by short TEG chain in the structure, showing less than one third uptake as compared to uncoated nanoMOFs (from 75% for uncoated nanoMOFs to 21% and 22% for nanoMOFs@**9** and nanoMOFs@**10**, respectively). This result suggests that the presence of short TEG chains optimizes the “stealth” effect with respect to longer PEG chains, which are not able to decrease very significantly the cellular uptake with respect to plain CD-P. Possibly, part of PEG chains might penetrate inside the nanoMOF pores, as previously reported [15]. Moreover, since the MW of PEG-CD **16** is much higher than that of **8**–**10**, and their coating efficiencies, which are based on P content in the coated samples, are similar, it was expected that the MOF surface would be coated with more molecules of **8**–**10** than molecules of **16**, and therefore the PEG density might not have been enough to promote a more efficient “stealth” effect.

In the cases of nanoMOFs@**8** and nanoMOFs@**10**, mannose grafting did not increase nanoMOFs uptake, as one would have expected. Macrophages internalize nanoparticles through different mechanisms depending on the physicochemical properties of the nanomaterials, including particle size, particle shape, surface charge, and surface modification with organic and/or biological molecules [66]. It is known that uncoated nanoMOFs uptake by J774A.1 macrophages is driven principally by phagocytosis [64], a mechanism which is strongly influenced by the specific composition of the protein corona [15,20,21]. “Stealth” effects of PEG coating on nanomaterials are not dependent on a reduction of protein corona adsorption but on a change of the type of proteins adsorbed on the surface from the blood plasma, more specifically to the binding of mainly clusterin protein [67]. Our results suggest that when mannose-coated nanoMOF@**8** and nanoMOF@**10** are placed in a complex biological environment, the binding of clusterin to the surface of such nanoparticles not only contributes to an efficient “stealth” effect, but also may shield the accessibility of the mannose ligands to their macrophage surface specific receptors. Furthermore, mannose-vectorized nanomaterials towards macrophages are dominantly internalized by energy-dependent clathrin-mediated endocytosis [68,69,70]. Such mechanism is affected by the particle size and works well when particles are smaller than 200 nm [71]. Thus, nanoMOFs@**8** and nanoMOFs@**10** uptake may also be hindered by the relatively large size of the nanoparticles. These observations reflect the complexity of surface functionalization of highly porous MIL-100(Fe) nanoMOFs.

### 3.5. DOX-Loaded MIL-100(Fe) nanoMOFs Surface Modification

Finally, we investigated the coating of anticancer drug DOX-loaded nanoMOF with TEGylated PCD **9**, since nanoMOF@**9** displayed the most efficient “stealth” effect. DOX payload reached 17 ± 3 wt%, corresponding to a loading efficiency of 85 ± 2%. This good loading efficiency indicated the strong interaction between DOX and MIL-100(Fe) nanoMOFs, in agreement with the previous reports [72]. The drug-loaded nanoMOFs were further coated with **9**, by the same one-step procedure in water described above. The functionalized particles had similar diameters (235 ± 21 nm) to those of the unloaded ones (226 ± 24 nm). Interestingly, surface modification of nanoMOFs with **9** did not induce DOX release, showing less than 2 ± 0.4% DOX release after 12 h incubation with this PCD. The coating of DOX-loaded nanoMOFs with targeting ligands and DOX release studies in cancer cells will be the subject of further studies. These results showed that the coating strategy described herein can be applied to nanoMOFs previously loaded with a drug.

## 4. Conclusions

In conclusion, we have developed a convenient method for the coating of nanoMOFs MIL-100(Fe) with TEG, PEG, and mannose residues. The method consisted of the organic solvent-free self-assembly on the nanoMOFs surface of building-blocks based on β-CD having functional moieties (TEG, PEG, and mannose) on one face and multiple phosphate groups on the other side. The coating can also be applied to doxorubicin-loaded nanoMOFs MIL-100(Fe) without significantly affecting the payload of the nanoparticles. Mannose-coated nanoMOFs displayed a remarkable enhanced binding affinity towards a specific mannose receptor such as Concanavalin A due to the so-called multivalent effect. However, these remarkable mannose recognition properties were not translated into improved macrophage uptake. By contrast, such mannosylated systems showed reduced macrophage internalization as compared with uncoated nanoMOFs. Furthermore, our results showed that coating nanoMOFs with cyclodextrin phosphate led to a significant “stealth” effect, slightly improved by PEGylated or mannosylated cyclodextrin phosphate coating. More notable, the nanoMOFs surface modification with TEGylated and mannosyl-TEGylated PCDs, having a much shorter oligoethylene chain, displayed the most efficient “stealth” effect. These results highlight the complexity of the involved interactions, particularly in a biological environment. Further research will be performed to comprehensively understand the localization and functions of each cyclodextrin phosphate component as well as the structural requirements to ensure molecular recognition properties in a biological medium. Our results showed that PCDs offer a versatile platform to coat nanoMOFs in an organic solvent-free, one step manner, and to impart biorecognition properties to nanoMOFs.

## Figures and Tables

**Table 1:** Thermodynamics and stability constants for the binding of conjugates **8**, **10**, nanoMOFs@**8**, and nanoMOFs@**10** to ConA according to an *n* equal and independent binding sites model.

Conjugate	*N ^a^*	*K* × 10^−5^ (M^−1^)	Δ*G*^0^ (kcal mol^−1^)	Δ*H* (kcal mol^−1^)	*T*Δ*S*^0^ (kcal mol^−1^)
**8** *^b^*	2.44 ± 0.01	2.25 ± 0.08	−7.30 ± 0.02	−14.49 ± 0.06	−7.19
**10** *^b^*	2.79 ± 0.01	5.12 ± 0.25	−7.79 ± 0.03	−13.96 ± 0.07	−6.17
nanoMOFs@**8** *^c^*	0.90 ± 0.01	14.20 ± 1.64	−8.39 ± 0.07	−334.20 ± 6.91	−325.81
nanoMOFs@**10** *^c^*	1.06 ± 0.01	24.50 ± 1.17	−8.72 ± 0.03	−314.10 ± 3.37	−305.38

*^a^* [conjugate]:[ConA] ratio when the lectin binding sites are fully saturated. Concentration of ConA was calculated as tetramer; *^b^* 20 mM phosphate buffer (pH 7.2) at 25 °C; *^c^* 10 mM TRIS buffer (pH 7.5) at 25 °C.

## Supplementary Materials

The following are available online at https://www.mdpi.com/2079-4991/9/8/1103/s1, Figure S1: ^1^H NMR spectrum (500 MHz, CDCl_3_, 25 °C) for compound **3**, Figure S2: ^1^H NMR spectrum (500 MHz, DMSO-*d*_6_, 80 °C) for compound **5**, Figure S3: ^1^H NMR spectrum (500 MHz, DMSO-*d*_6_, 80 °C) for compound **6**, Figure S4: ^1^H NMR spectrum (500 MHz, DMSO-*d*_6_, 80 °C) for compound **7**, Figure S5: ^1^H NMR spectrum (600 MHz, D_2_O, 25 °C) for compound **8**, Figure S6: ^1^H NMR spectrum (600 MHz, D_2_O, 25 °C) for compound **9**, Figure S7: ^1^H NMR spectrum (600 MHz, D_2_O, 25 °C) for compound **10**, Figure S8: ^1^H NMR spectrum (300 MHz, D_2_O, 25 °C) for compound **14**, Figure S9: ^1^H NMR spectrum (600 MHz, D_2_O, 25 °C) for compound **15**, Figure S10: ^1^H NMR spectrum (600 MHz, D_2_O, 25 °C) for compound **16**, Figure S11: ^13^C NMR spectrum (125 MHz, CDCl_3_, 25 °C) for compound **3**, Figure S12: ^13^C NMR spectrum (125 MHz, DMSO-*d*_6_, 80 °C) for compound **5**, Figure S13: ^13^C NMR spectrum (125 MHz, DMSO-*d*_6_, 80 °C) for compound **6**, Figure S14: ^13^C NMR spectrum (125 MHz, DMSO-*d*_6_, 80 °C) for compound **7**, Figure S15: ^13^C NMR spectrum (150 MHz, D_2_O, 25 °C) for compound **8**, Figure S16: ^13^C NMR spectrum (150 MHz, D_2_O, 25 °C) for compound **9**, Figure S17: ^13^C NMR spectrum (150 MHz, D_2_O, 25 °C) for compound **10**, Figure S18: ^13^C NMR spectrum (75 MHz, D_2_O, 25 °C) for compound **14**, Figure S19: ^13^C NMR spectrum (150 MHz, D_2_O, 25 °C) for compound **15**, Figure S20: ^31^P NMR spectrum (242.9 MHz, D_2_O, 25°C) for compound **8**, Figure S21: ^31^P NMR spectrum (242.9 MHz, D_2_O, 25 °C) for compound **9**, Figure S22: ^31^P NMR spectrum (242.9 MHz, D_2_O, 25 °C) for compound **10**, Figure S23: ^31^P NMR spectrum (242.9 MHz, D_2_O, 25 °C) for compound **16**, Table S1: Determination of the number of phosphate groups for compounds **10**–**12** and **18**, Figure S24: XRPD patterns of uncoated nanoMOFs and nanoMOFs coated with CD-P, **8**–**10**, and **16**, Figure S25: DLS size measurements of uncoated nanoMOFs and nanoMOFs coated with CD-P, **8**-**10** and **16** after 6 h incubation in DMEM cell culture medium complemented with 10% FBS., Figure S26: PCDs derivatives grafting efficiency versus nanoMOFs:PCD mass ratio assayed, Table S2: ITC experiments for the interaction of **8**, **10**, nanoMOFs@**8**, and nanoMOFs@**10** with Concanavalin A, Figure S27: Titration of ConA with conjugates **8** and **10** in 20 mM phosphate buffer (pH 7.2) at 25 °C, and titration of conjugates nanoMOFs@**8** and nanoMOFs@**10** with ConA in 10 mM TRIS buffer (pH 7.5) at 25 °C, Figure S28: Enthalpy-entropy compensation for the interaction of **8**, **10**, nanoMOFs@**8**, and nanoMOFs@**10** with ConA lectin at 25 °C.

## Abbreviations

β-CD	β-cyclodextrin
CD-P	β-cyclodextrin phosphate sodium salt
ConA	concanavalin A
DMAP	4-dimethylaminopyridine
DMEM	Dulbecco’s modified Eagle’s medium
DMF	dimethylformamide
DOX	doxorubicin
EtOAc	ethyl acetate
FBS	fetal bovine serum
HR-ICP-MS	high resolution inductively couples plasma mass spectrometry
ITC	isothermal titration calorimetry
MALDI-TOF-MS	matrix-assisted laser desorption/ionization time-of-flight mass spectrometry
MWCO	molecular weight cut-off
NaAsc	(+)-sodium l-ascorbate
nanoMOFs	nanosized metal-organic frameworks
PCDs	phosphorylated β-CD derivatives
PEG	polyethylene glycol
TEG	tetraethylene glycol
TEM	transmission electron microscopy
TLC	thin-layer chromatography
XRDP	X-ray diffraction patterns
ZP	zeta potential
