# Supplementary material for: Design of Engineered Cyclodextrin Derivatives for Spontaneous Coating of Highly Porous Metal-Organic Framework Nanoparticles in Aqueous Media
Source: Nanomaterials (Basel). 2019 Aug 1;9(8):1103. doi: 10.3390/nano9081103 (PMC6723150; doi:10.3390/nano9081103)
Supplement: Supplementary file 1 [file nanomaterials-09-01103-s001.pdf]

Design of Engineered Cyclodextrin Derivatives for Spontaneous Coating of Highly Porous Metal-Organic Framework Nanoparticles in Aqueous Media

Giovanna Cutrone [1], Xue Li [2], Juan M. Casas-Solvas [1], Mario Menendez-Miranda [2], Jingwen Qiu [2], Gabor Benkovics [3], Doru Constantin [4], Milo Malanga [3], Borja Moreira-Alvarez [5], José M. Costa-Fernández [5], Luis García-Fuentes [1], Ruxandra Gref [2,*] and Antonio Vargas-Berenguel [1,*]

[1] Department of Chemistry and Physics, University of Almería, Crta. de Sacramento s/n, E-04120, Almería, Spain
[2] Institut des Sciences Moléculaires d'Orsay, UMR CNRS 8214, Université Paris-Sud, Université Paris Saclay, 91400 Orsay, France
[3] CycloLab R&D Ltd, Illatos út 7, Budapest, H-1097, Hungary
[4] Laboratoire de Physique des Solides, UMR 8502, Université Paris-Sud, 91405 Orsay, France
[5] Department of Physical and Analytical Chemistry, University of Oviedo, Julián Clavería 8, 33006 Oviedo, Spain
∗ Corresponding author. E-mail address: ruxandra.gref@u-psud.fr (R. Gref); avargas@ual.es (A. Vargas-Berenguel)

Supplementary Materials

Table of Contents

I.	^{1}H NMR spectra for compounds **3**, **5-10** and **14-16**	S2-S11
II.	^{13}C NMR spectra for compounds **3**, **5-10** and **14-16**	S12-S20
III.	^{31}P NMR spectra for compounds **8-10** and **16**	S21-S24
IV.	Determination of the number of phosphate groups for compounds **8-10** and **16**	S25
V.	MIL-100(Fe) nanoMOFs synthesis and surface modification	S26-S27
VI.	ITC experiments for the interaction of **8**, **10**, nanoMOFs@**8** and nanoMOFs@**10** with Concanavalin A	S28-S29

I. ^{1}H-NMR spectra for compounds 3, 5-10 and 14-16

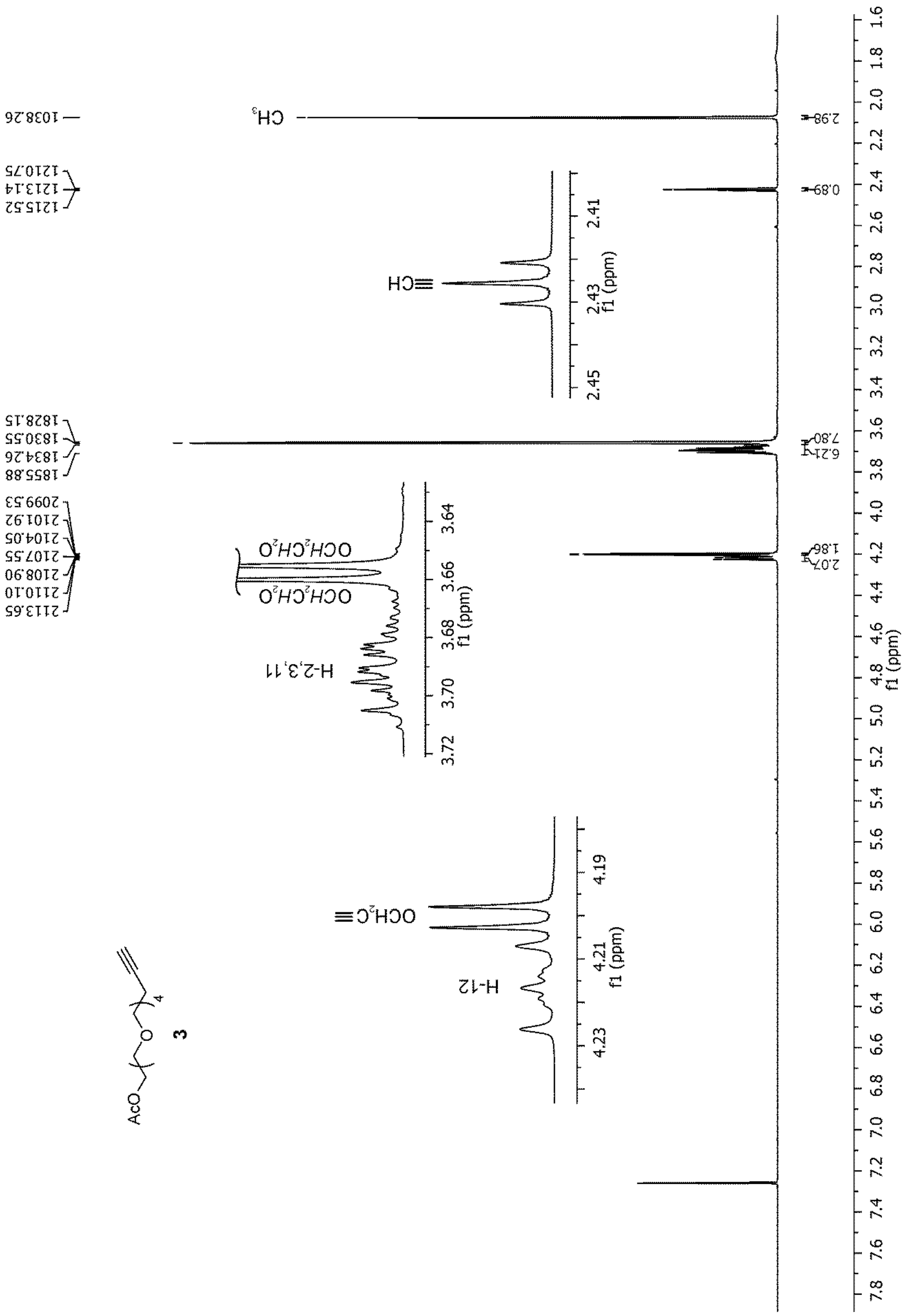

Figure S1. ^{1}H NMR spectrum (500 MHz, $CDCl_3$, 25 °C) for compound **3**

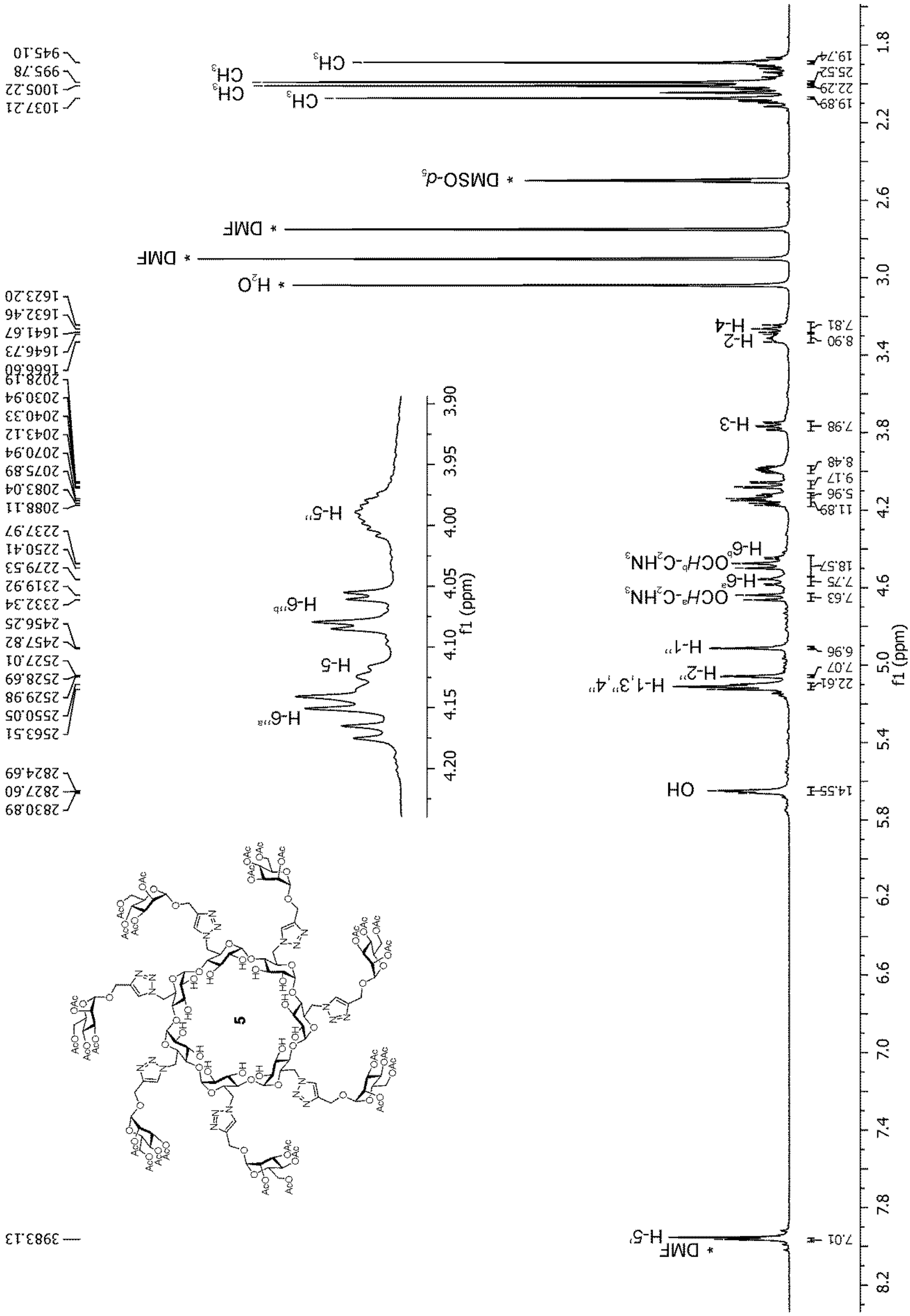

Figure S2. ^{1}H NMR spectrum (500 MHz, DMSO-d_6, 80 °C) for compound **5**

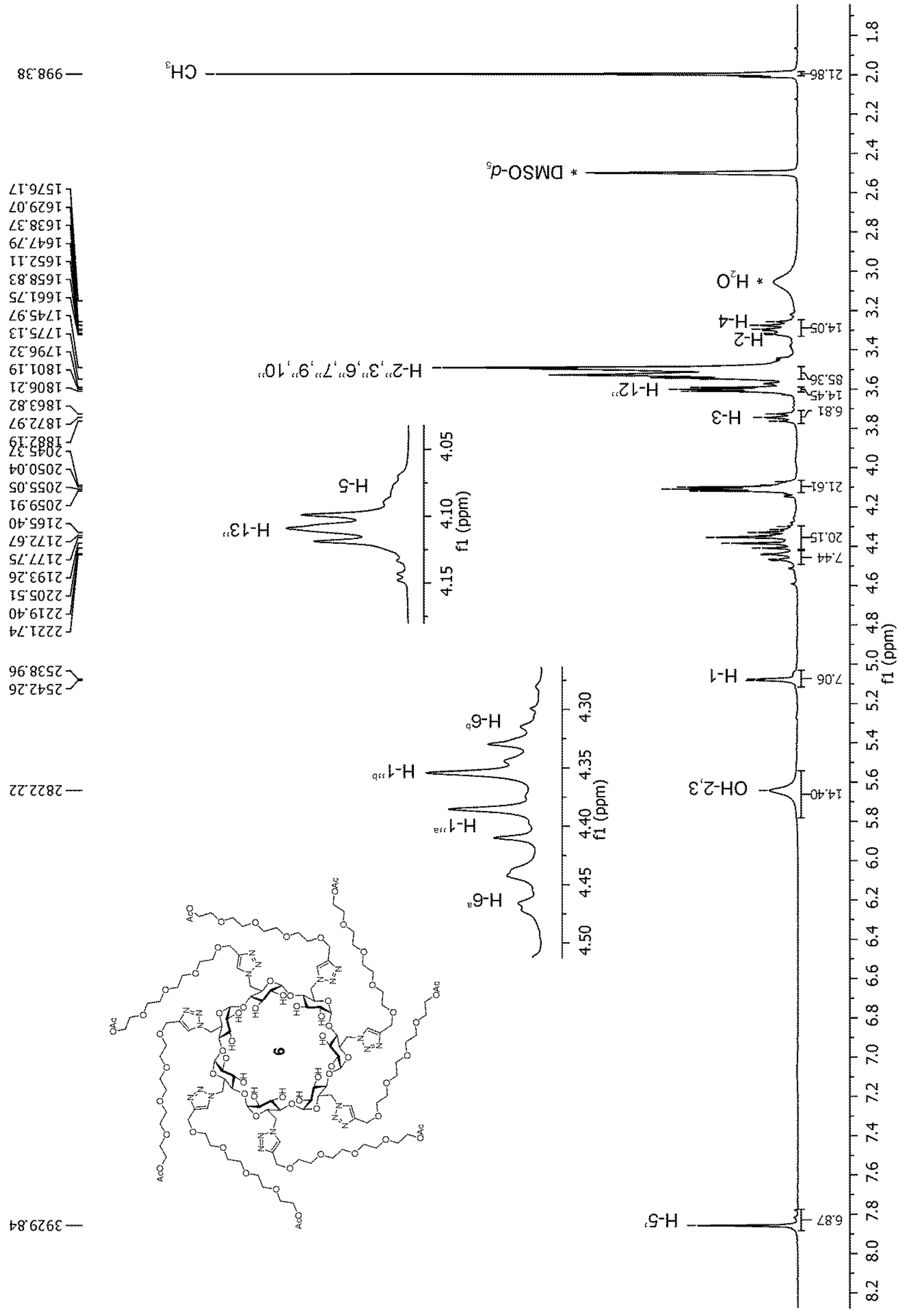

Figure S3. ^{1}H NMR spectrum (500 MHz, DMSO-d_6, 80 °C) for compound **6**

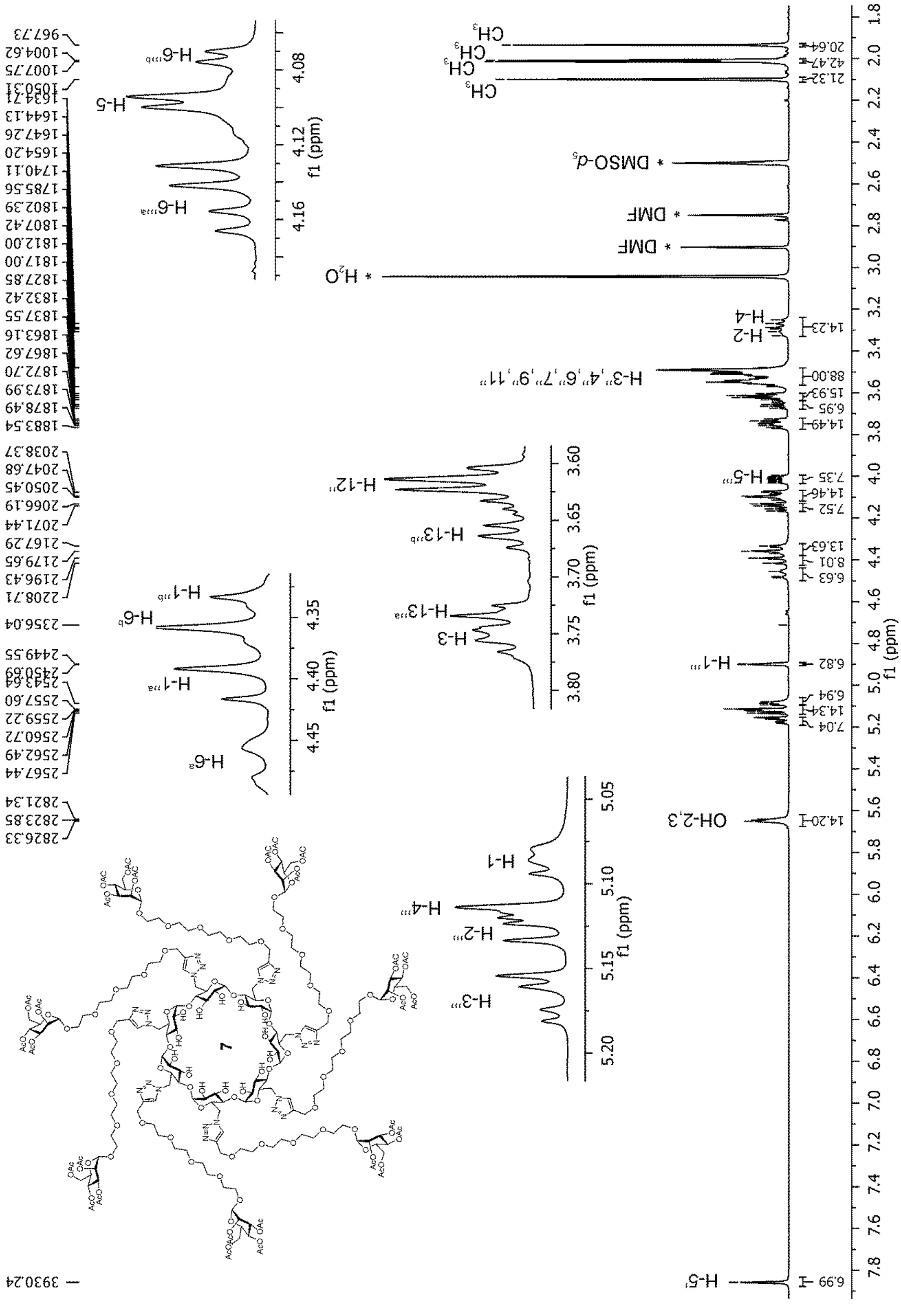

Figure S4. ^{1}H NMR spectrum (500 MHz, DMSO-d_6, 80 °C) for compound **7**

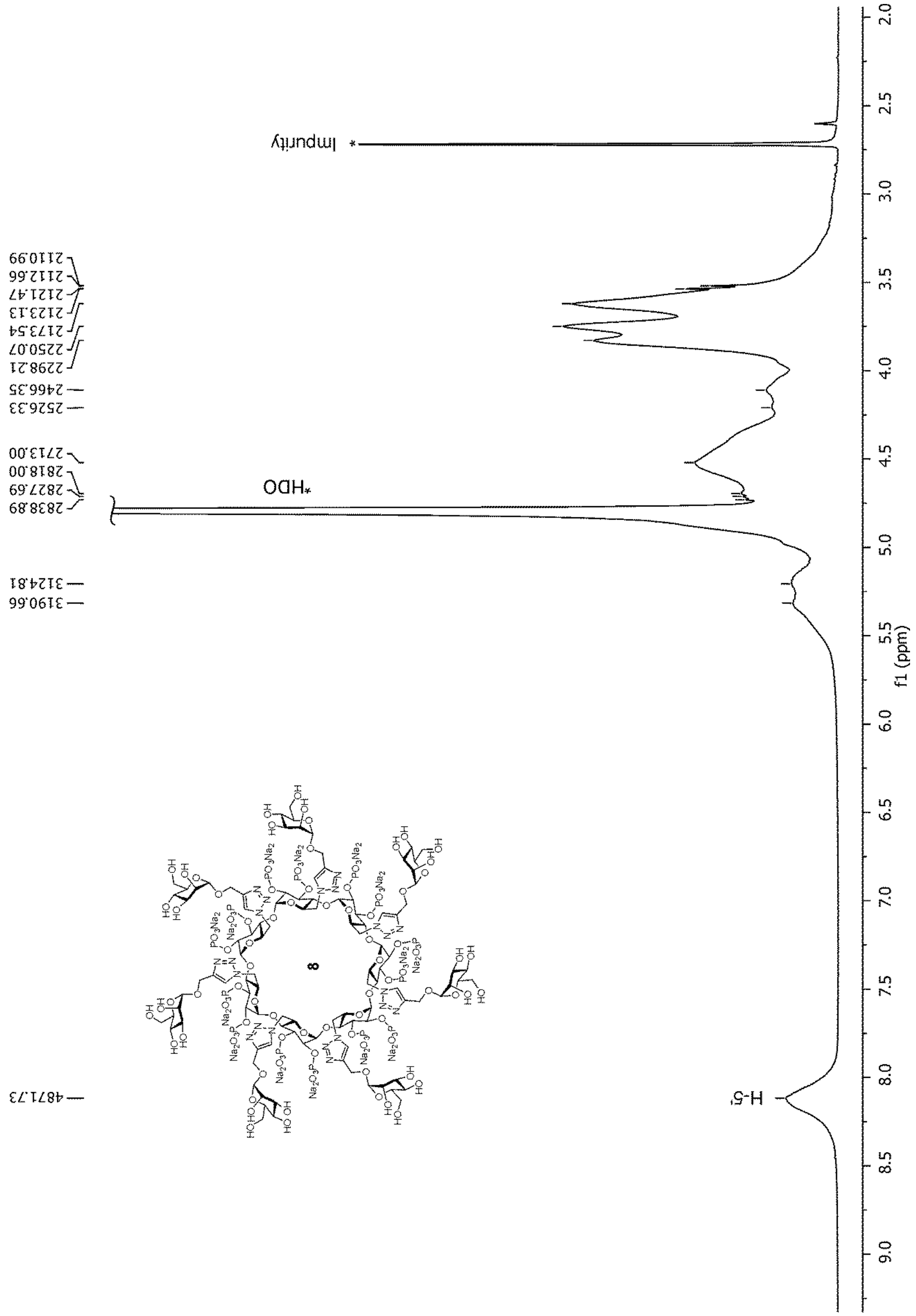

Figure S5. ^{1}H NMR spectrum (600 MHz, D_2O, 25 °C) for compound **8**

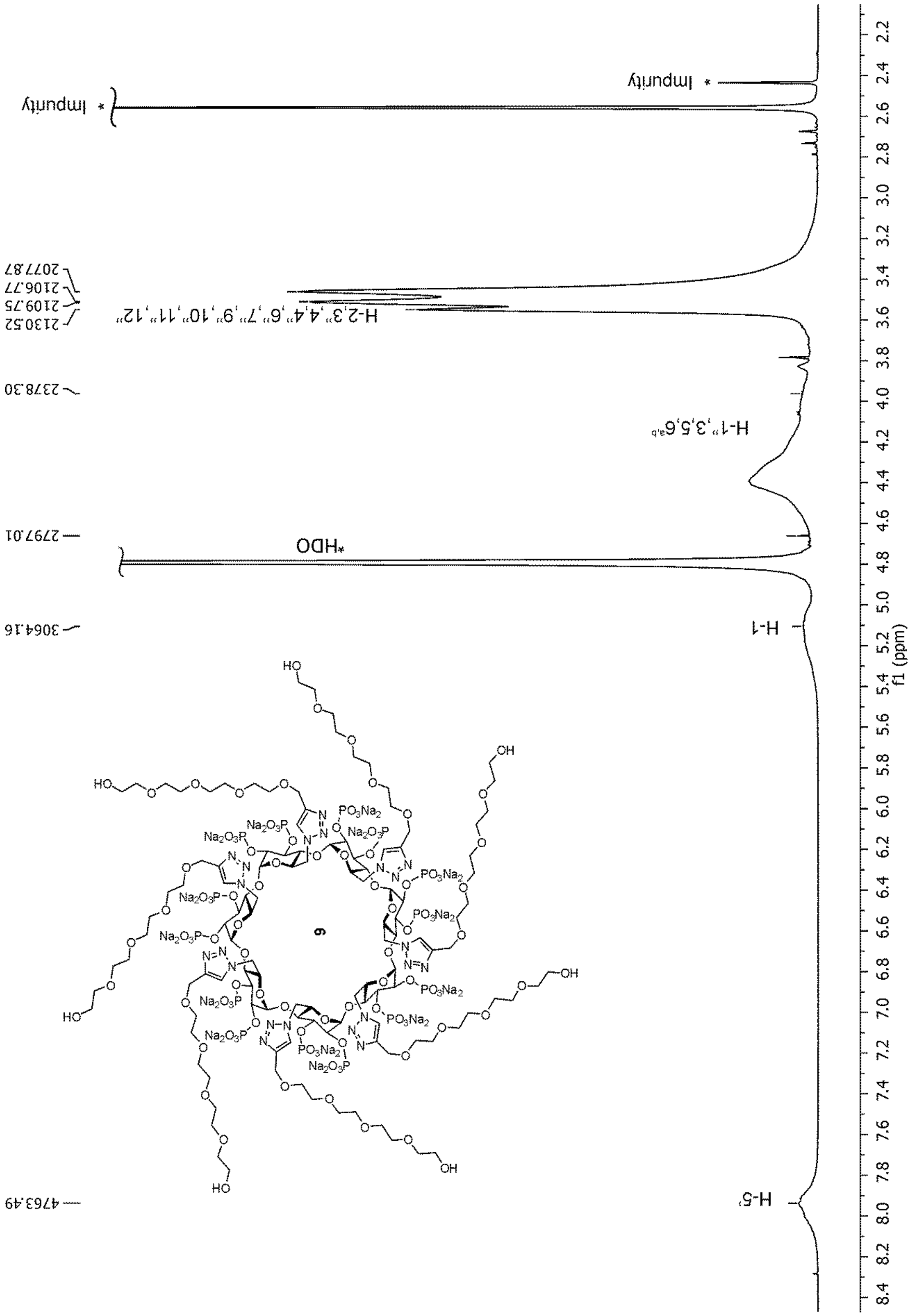

Figure S6. ^{1}H NMR spectrum (600 MHz, D_2O, 25 °C) for compound **9**

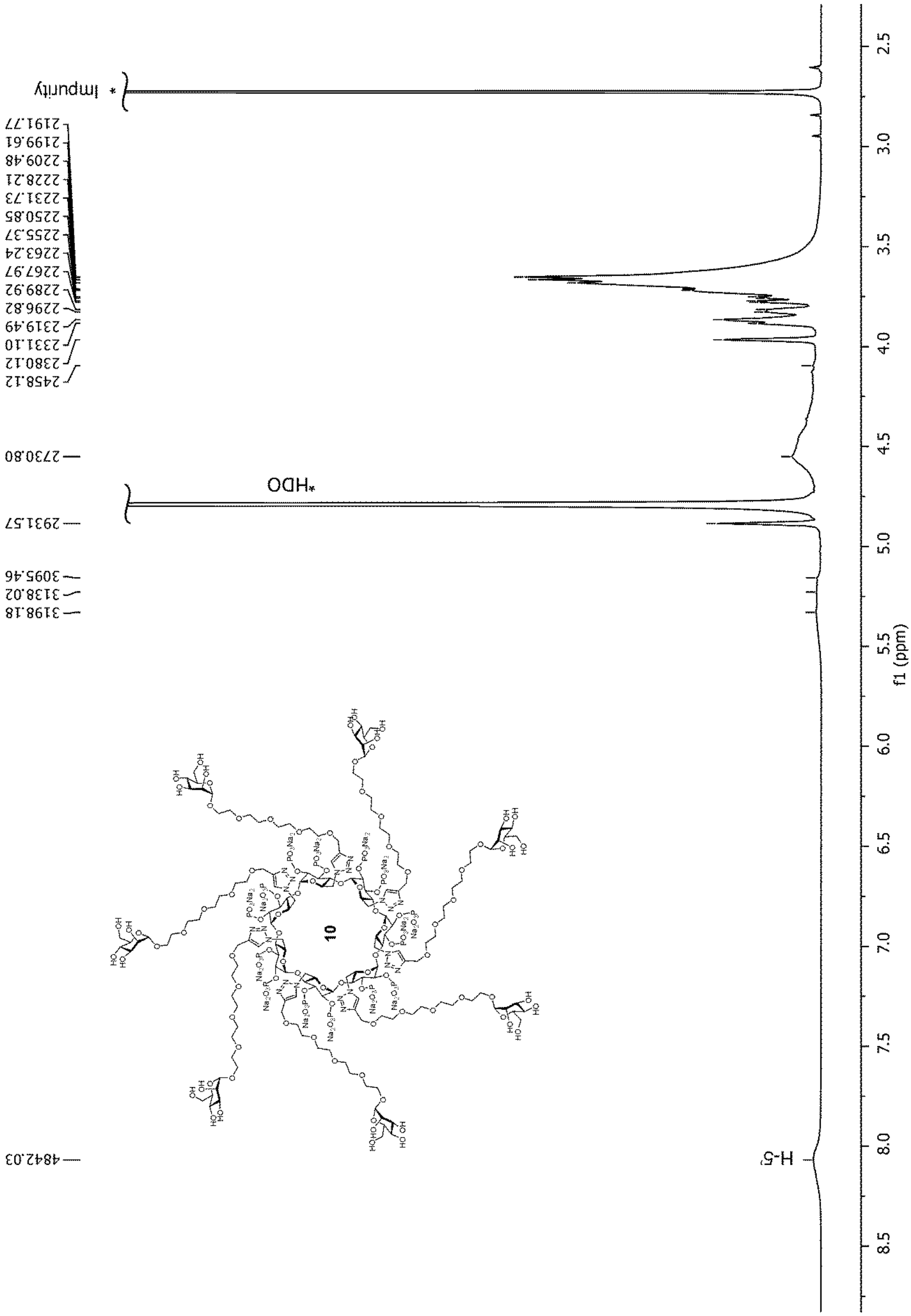

Figure S7. ^{1}H NMR spectrum (600 MHz, D_2O, 25 °C) for compound **10**

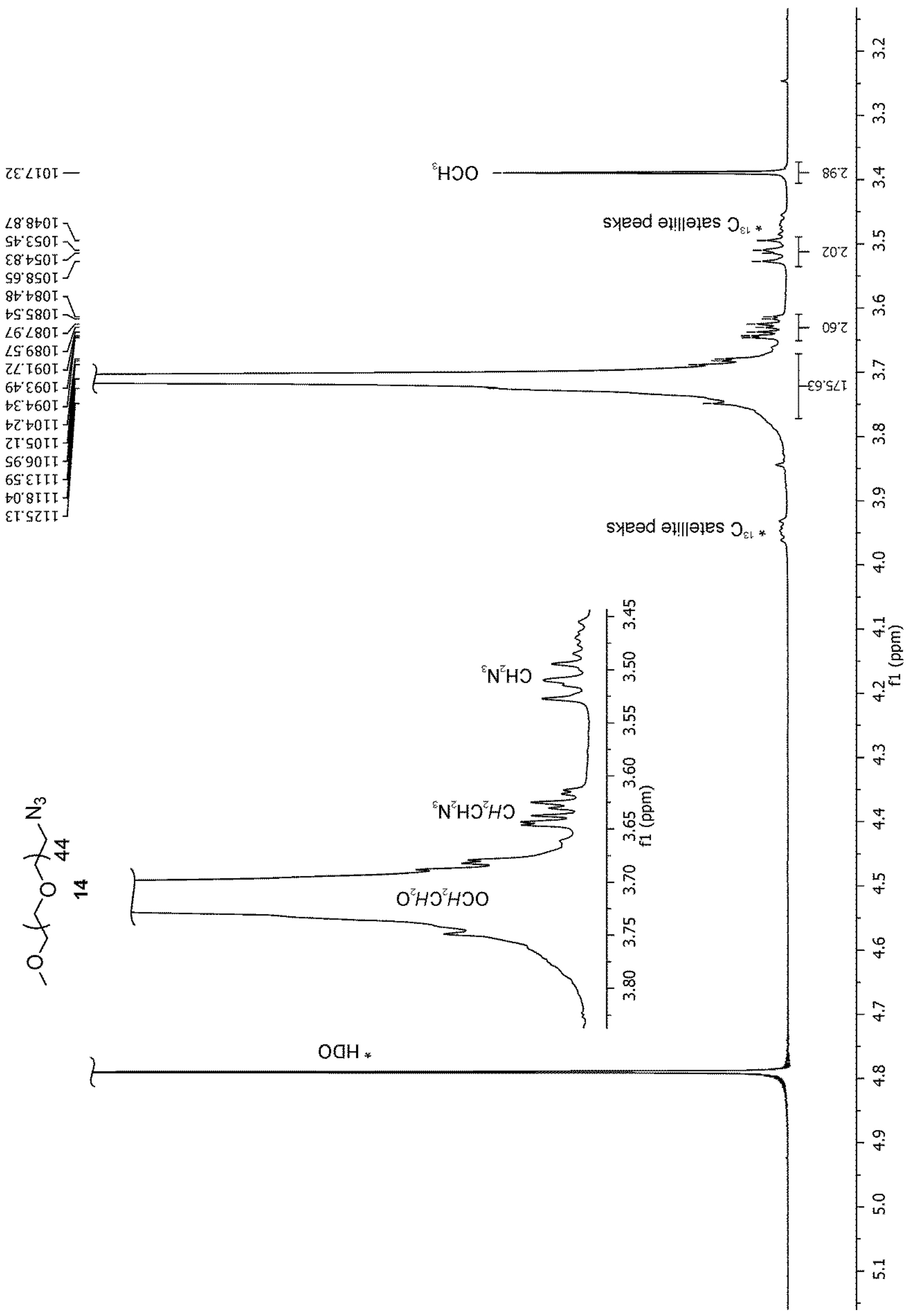

Figure S8. ^{1}H NMR spectrum (300 MHz, D_2O, 25 °C) for compound **14**

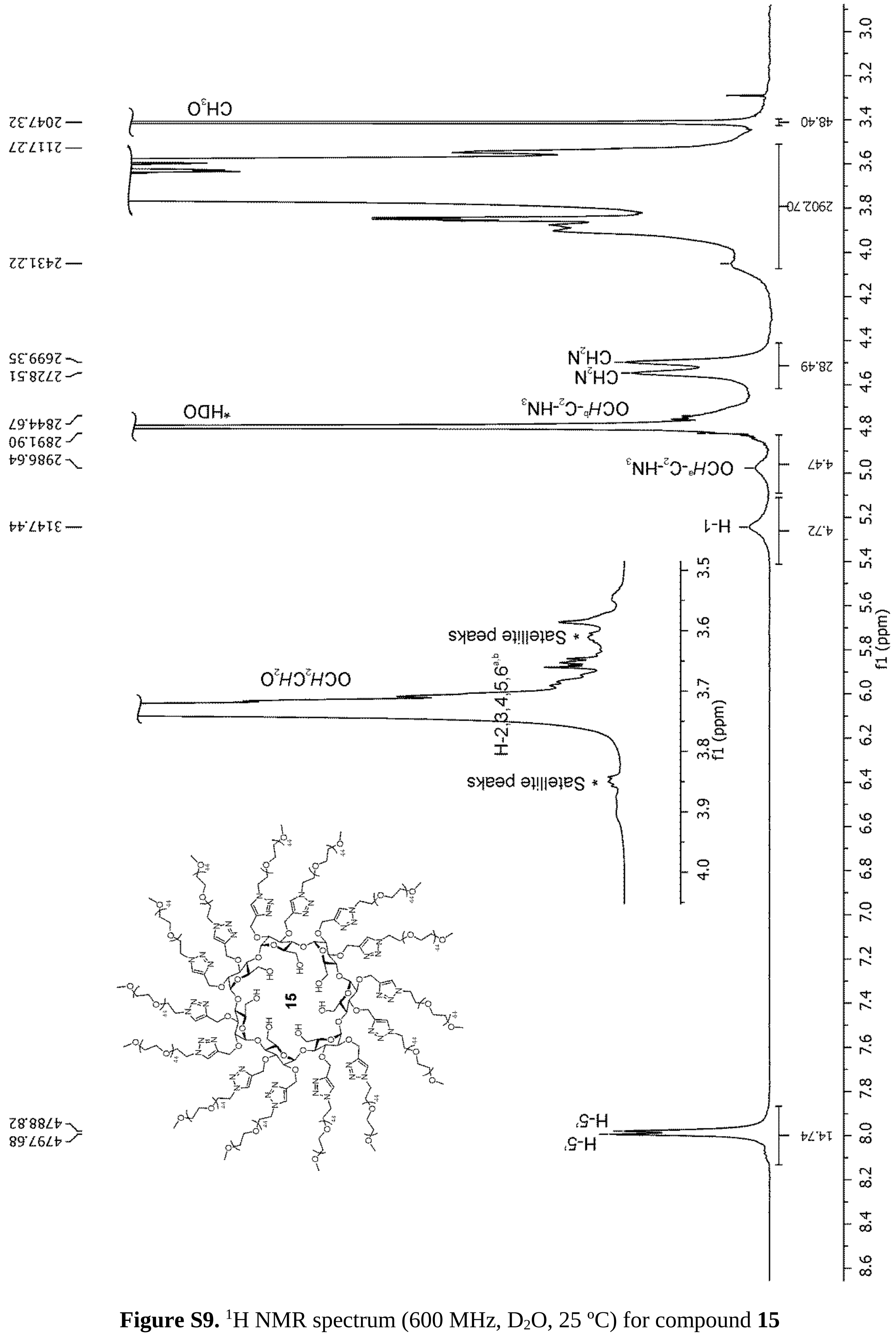

Figure S9. ^{1}H NMR spectrum (600 MHz, D_2O, 25 °C) for compound **15**

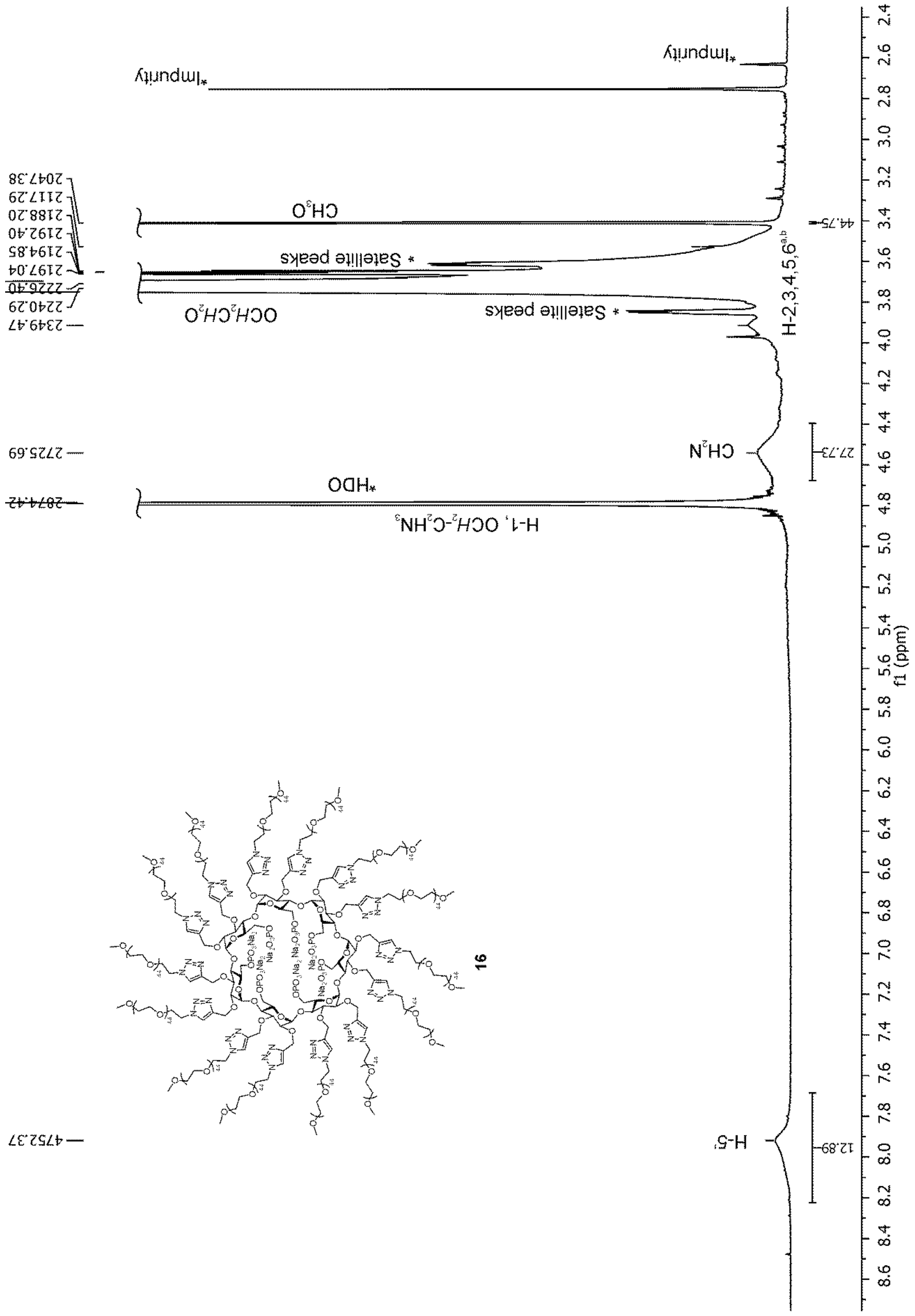

Figure S10. ^{1}H NMR spectrum (600 MHz, D_2O, 25 °C) for compound **16**

II. ^{13}C-NMR spectra for compounds 3, 5-10 and 14-16

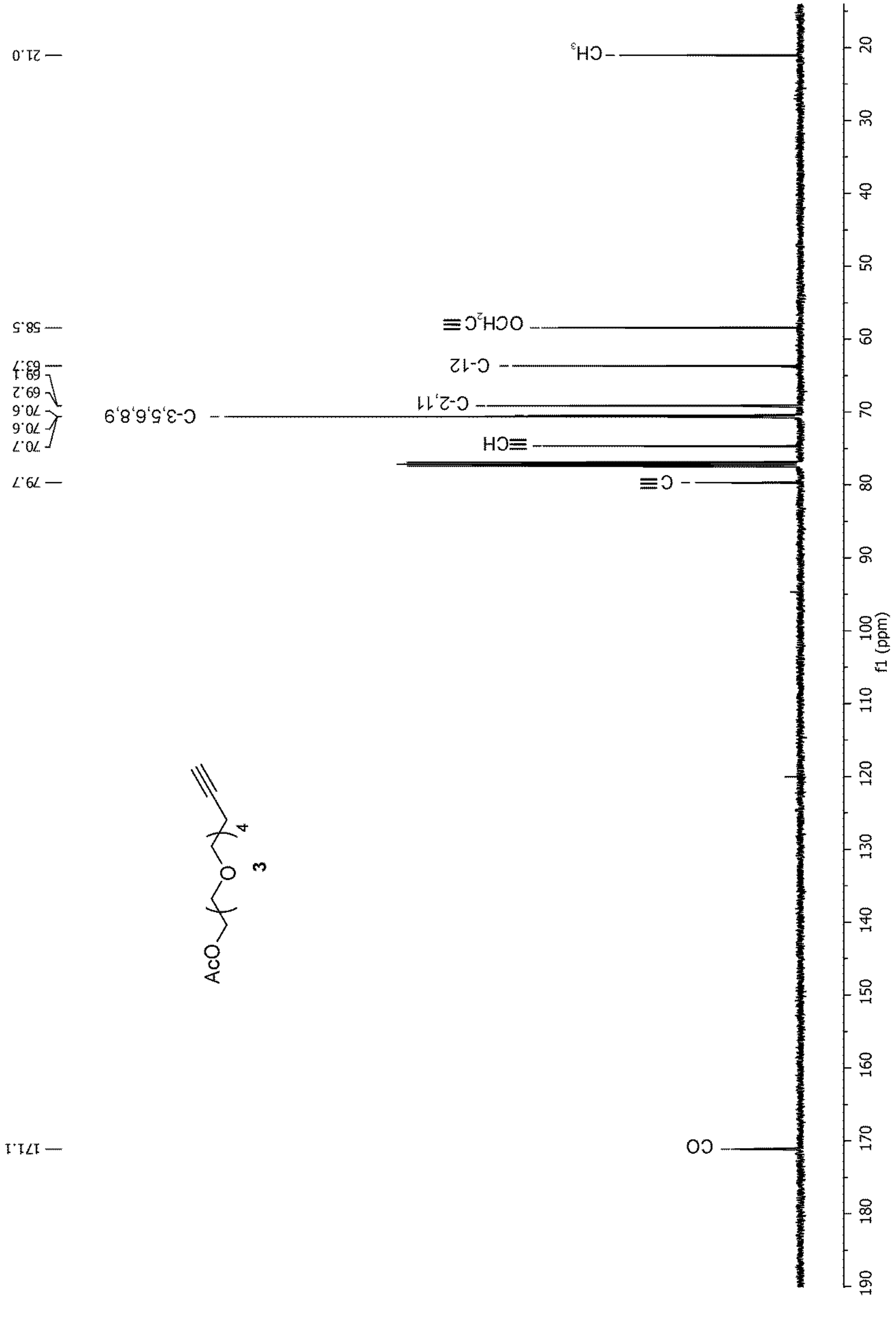

Figure S11. ^{13}C NMR spectrum (125 MHz, $CDCl_3$, 25 °C) for compound **3**

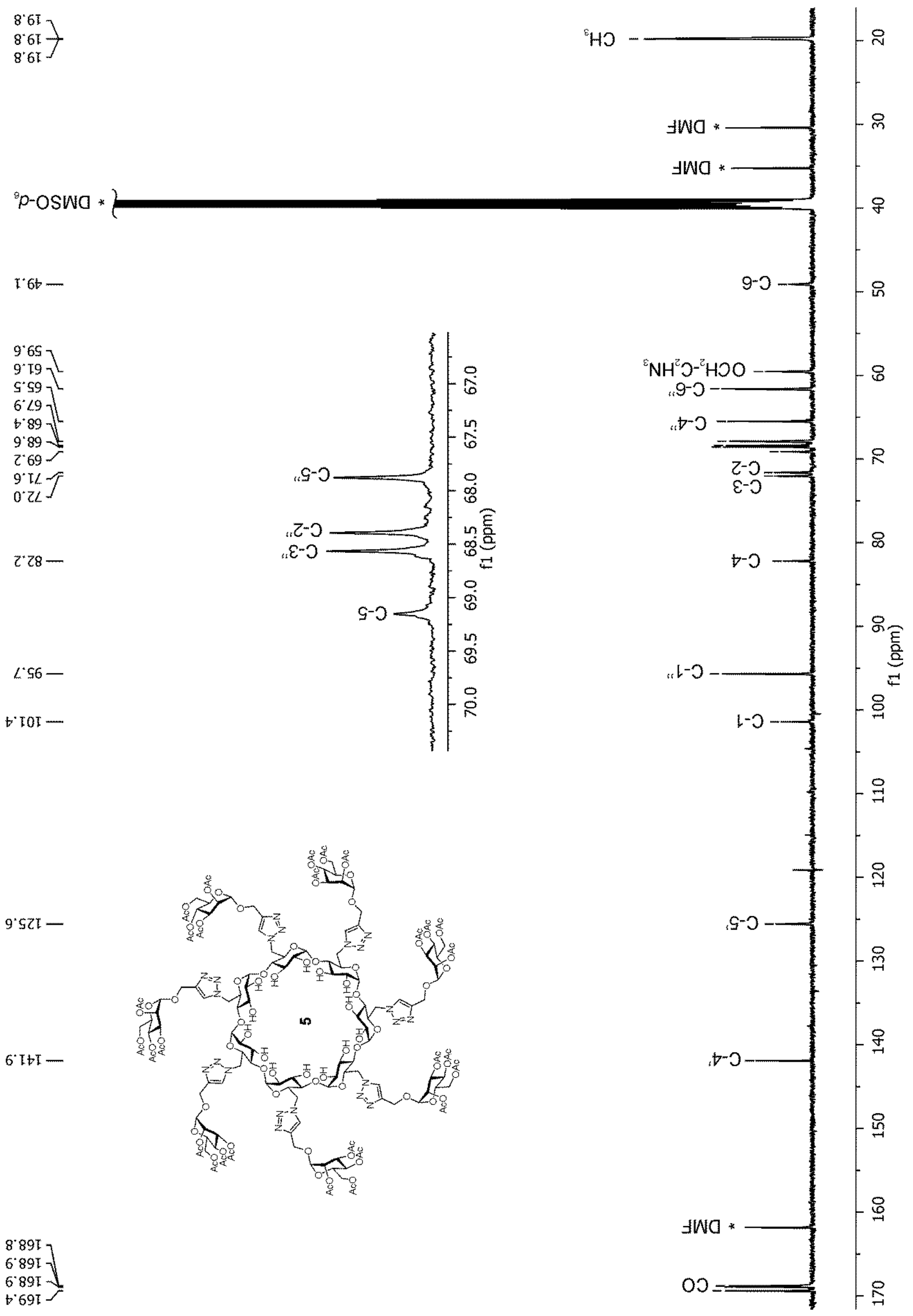

Figure S12. ^{13}C NMR spectrum (125 MHz, DMSO-d_6, 80 °C) for compound **5**

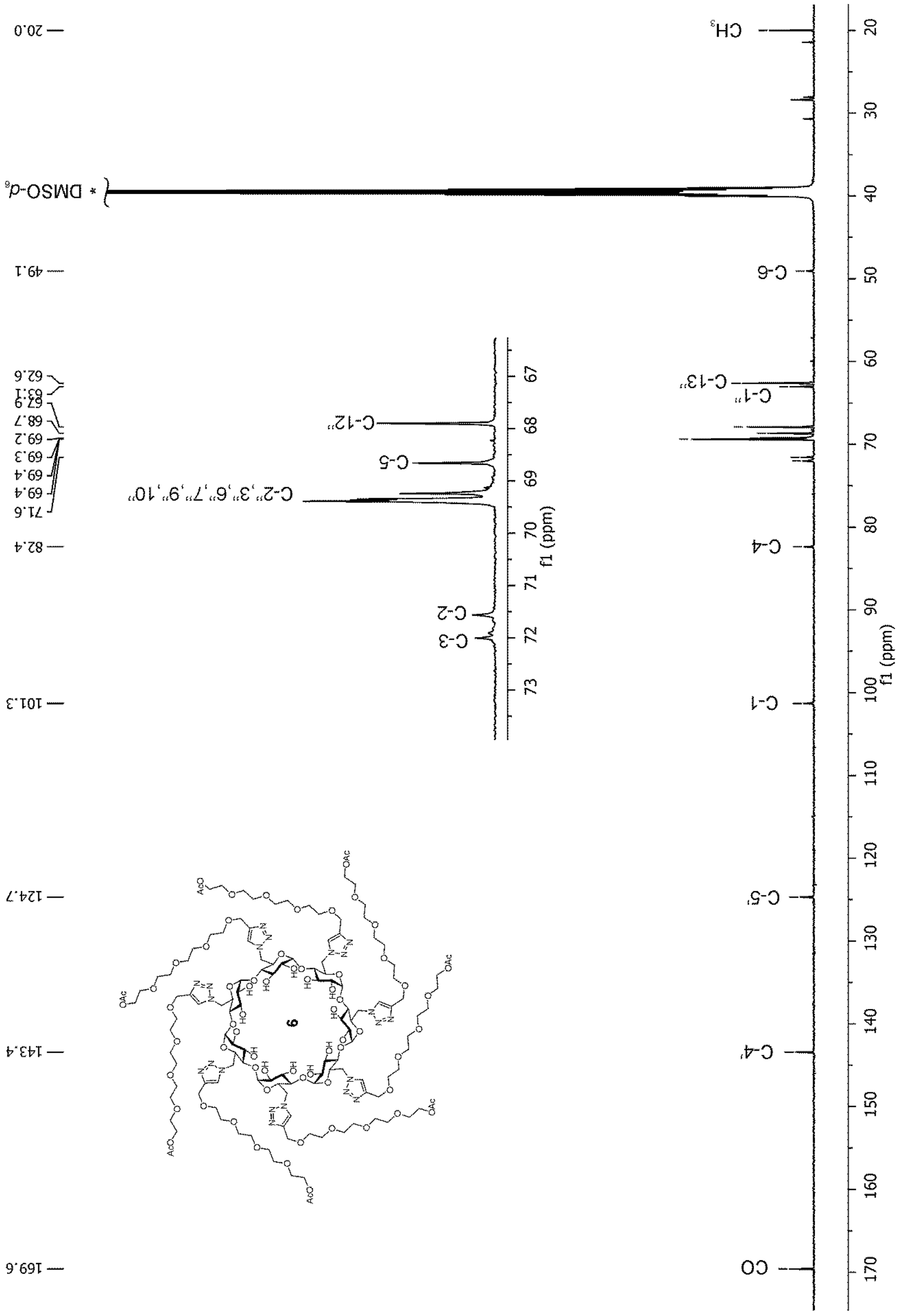

Figure S13. ^{13}C NMR spectrum (125 MHz, DMSO-d_6, 80 °C) for compound **6**

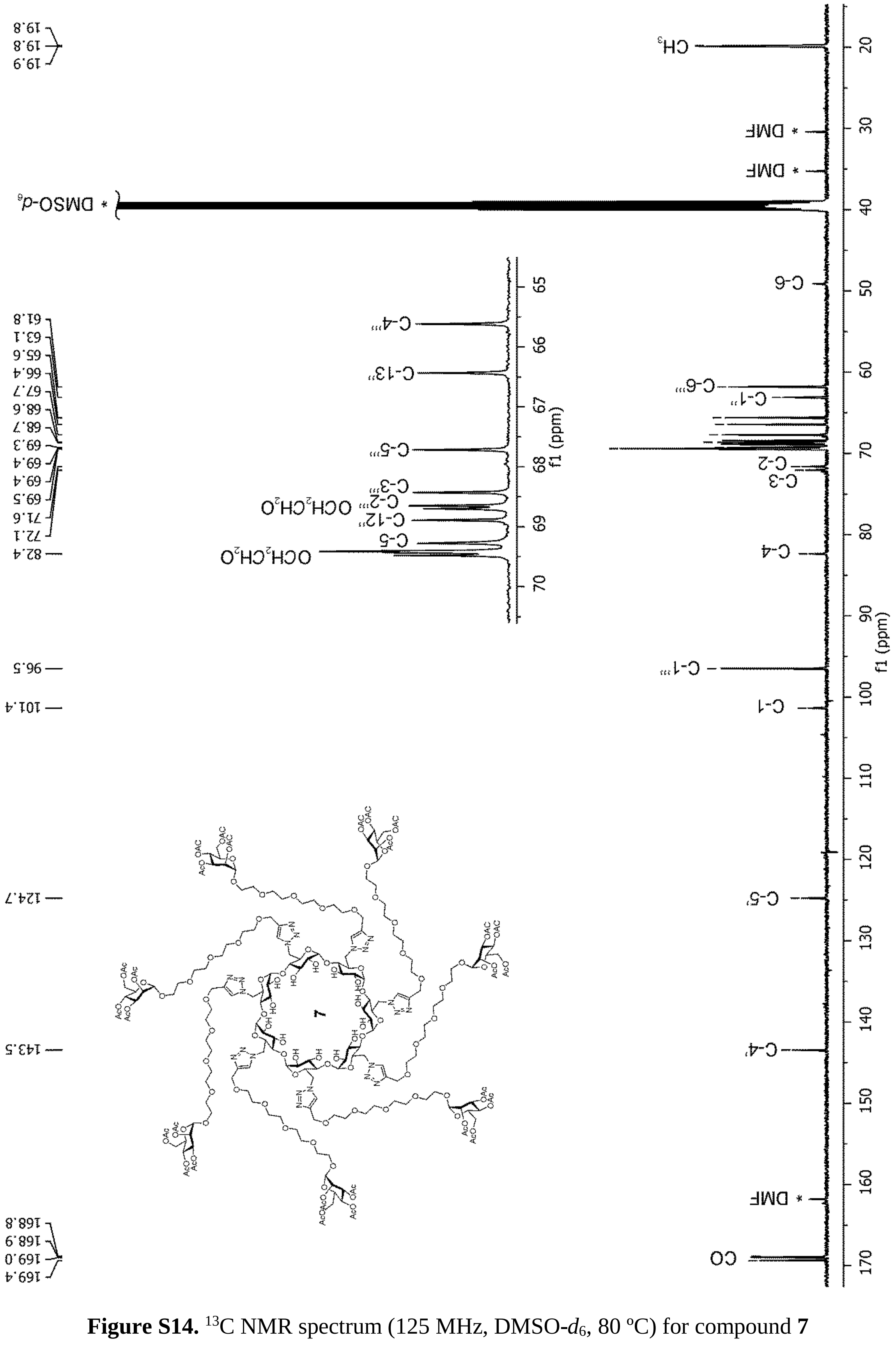

Figure S14. ^{13}C NMR spectrum (125 MHz, DMSO-d_6, 80 °C) for compound **7**

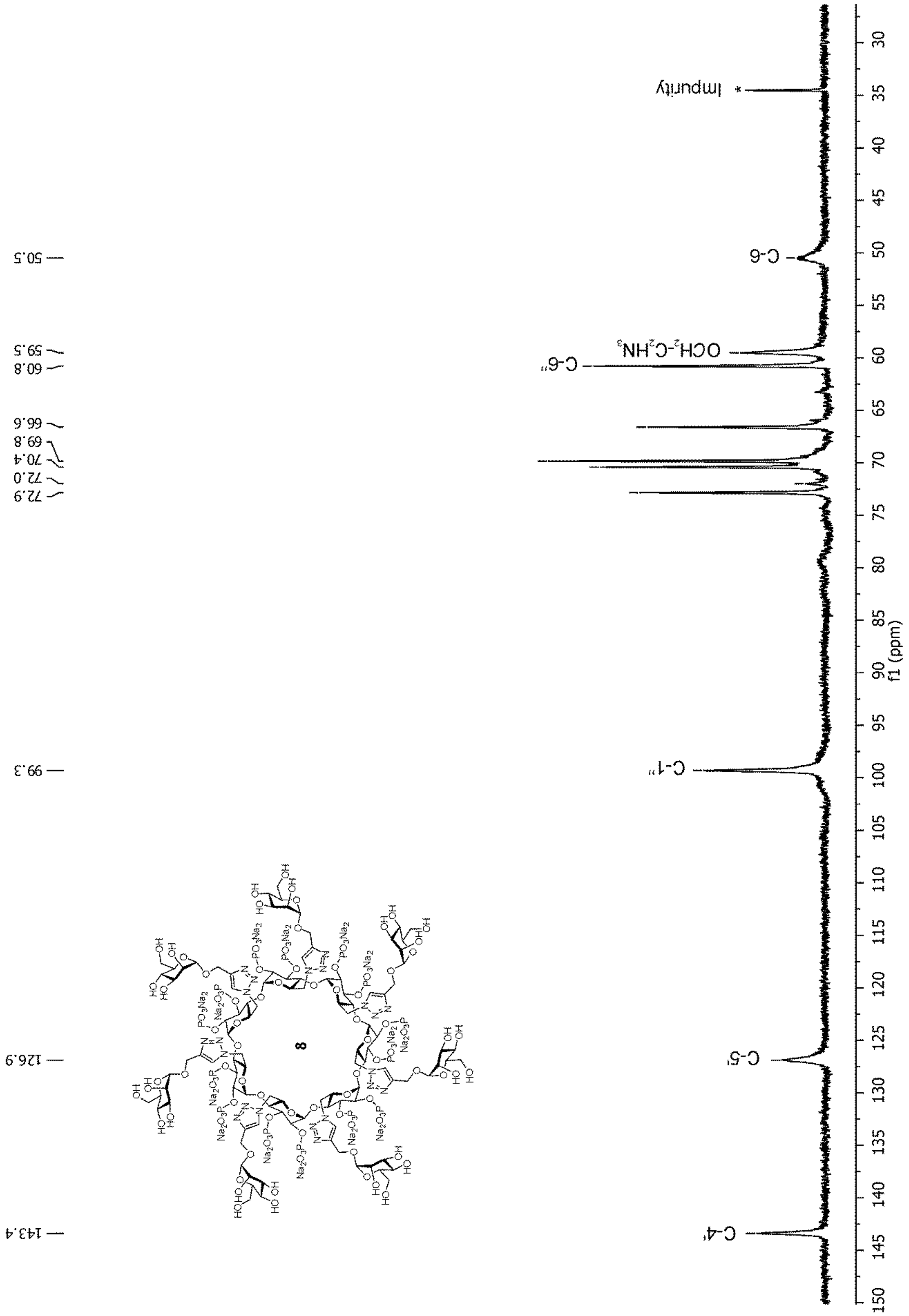

Figure S15. ^{13}C NMR spectrum (150 MHz, D_2O, 25 ºC) for compound **8**

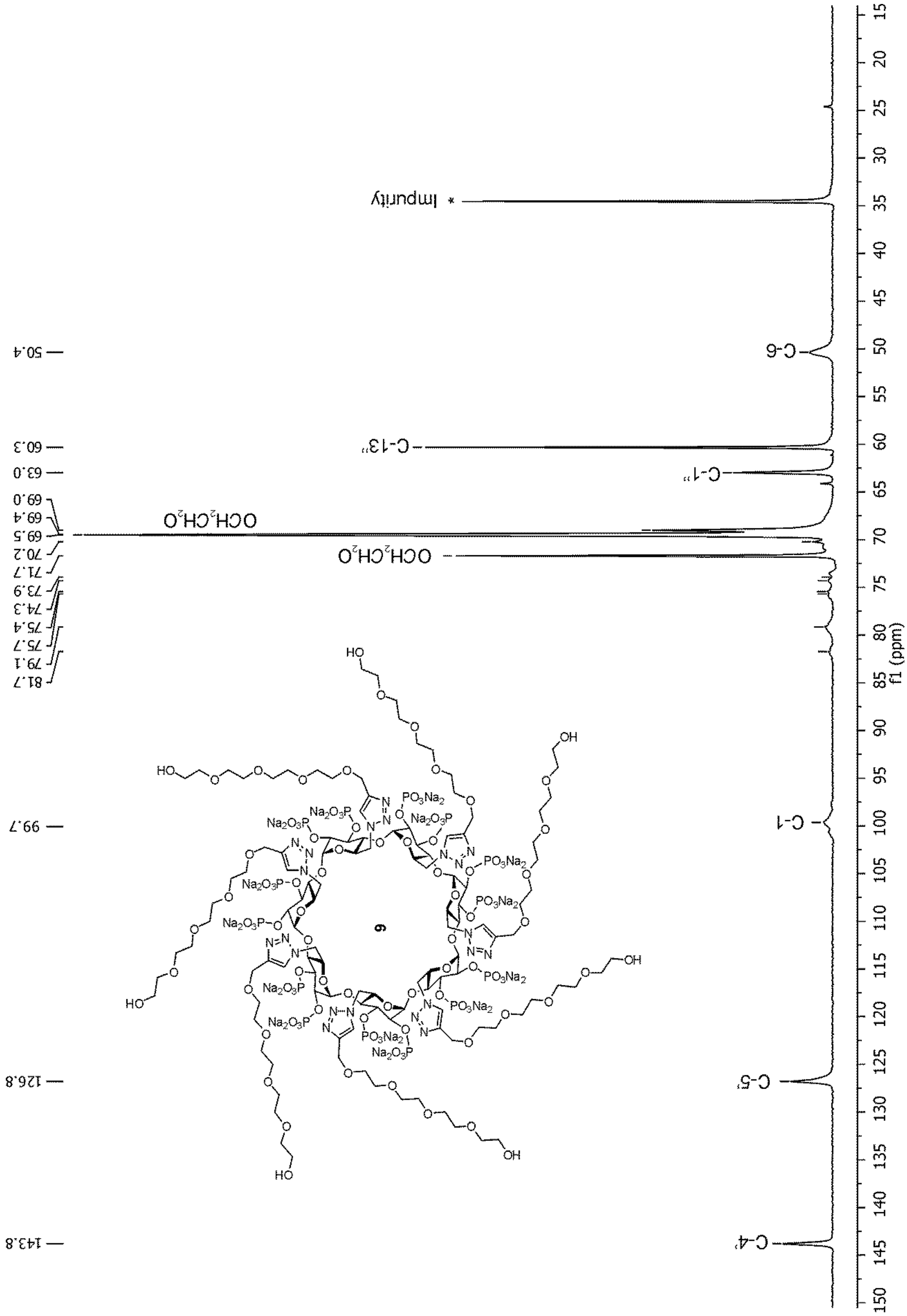

Figure S16. ^{13}C NMR spectrum (150 MHz, D_2O, 25 ºC) for compound **9**

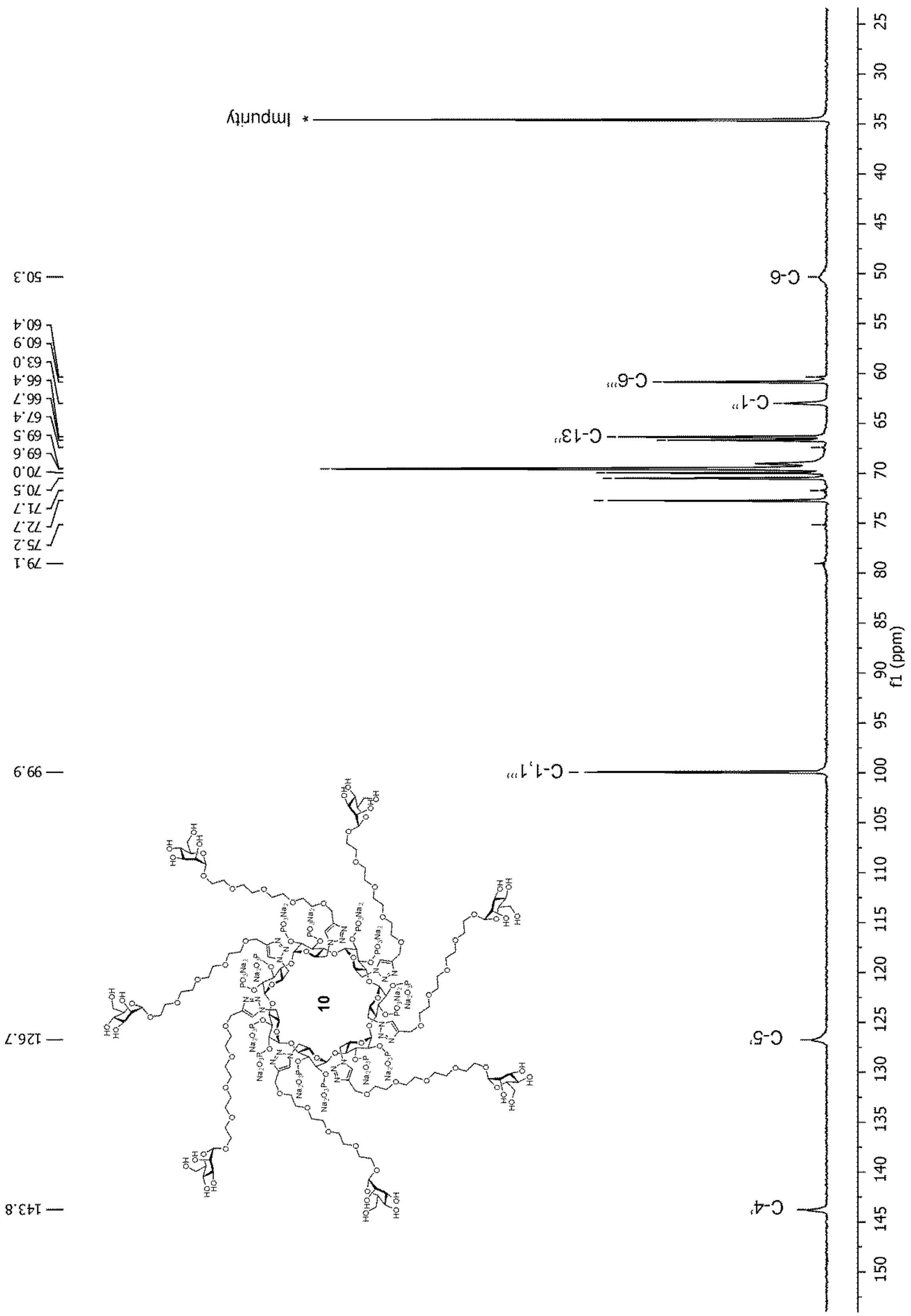

Figure S17. ^{13}C NMR spectrum (150 MHz, D_2O, 25 °C) for compound **10**

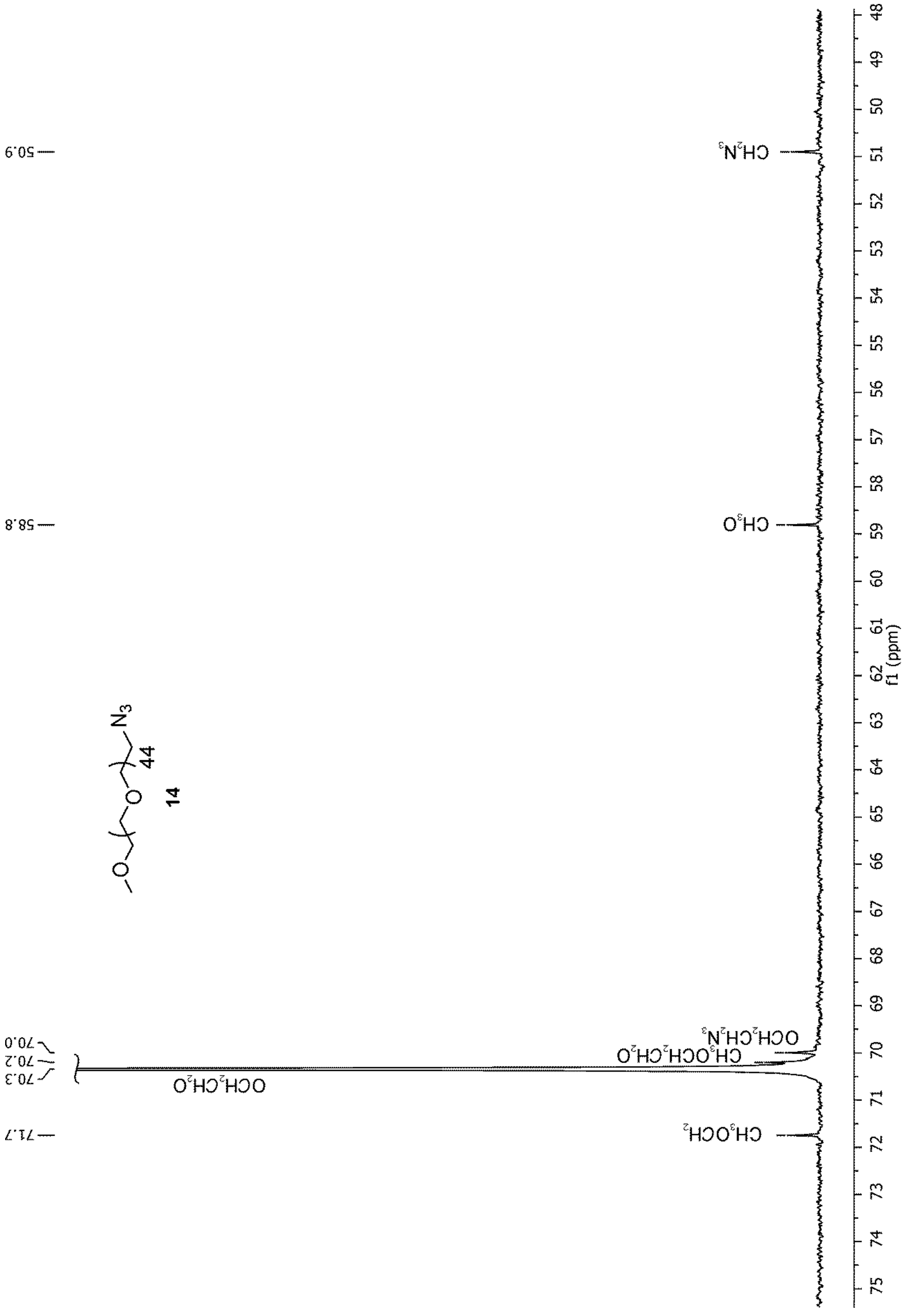

Figure S18. ^{13}C NMR spectrum (75 MHz, D_2O, 25 °C) for compound **14**

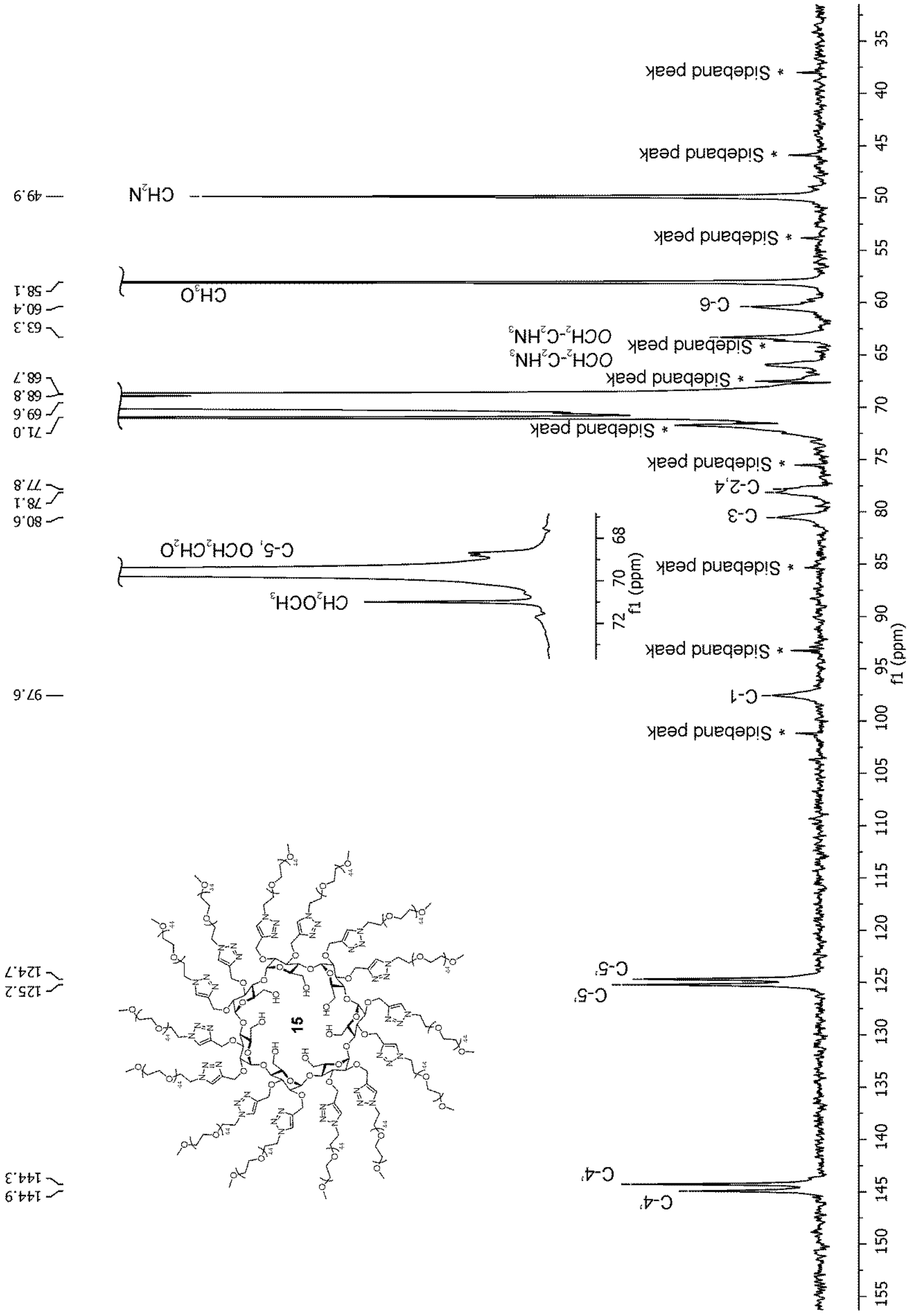

Figure S19. ^{13}C NMR spectrum (150 MHz, D_2O, 25 °C) for compound **15**

III. ^{31}P-NMR spectra for compounds 8-10 and 16

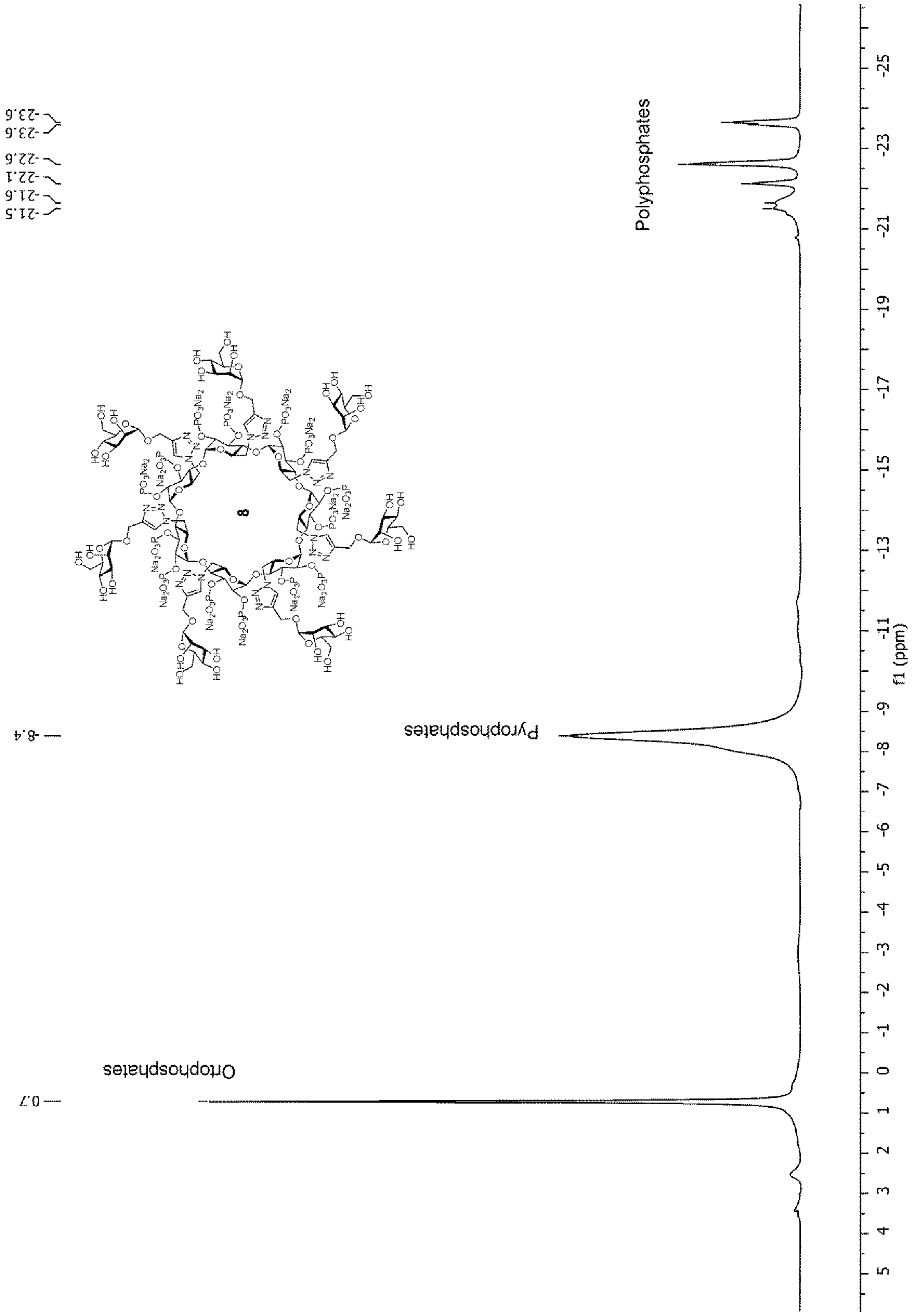

Figure S20. ^{31}P NMR spectrum (242.9 MHz, D_2O, 25 °C) for compound **8**

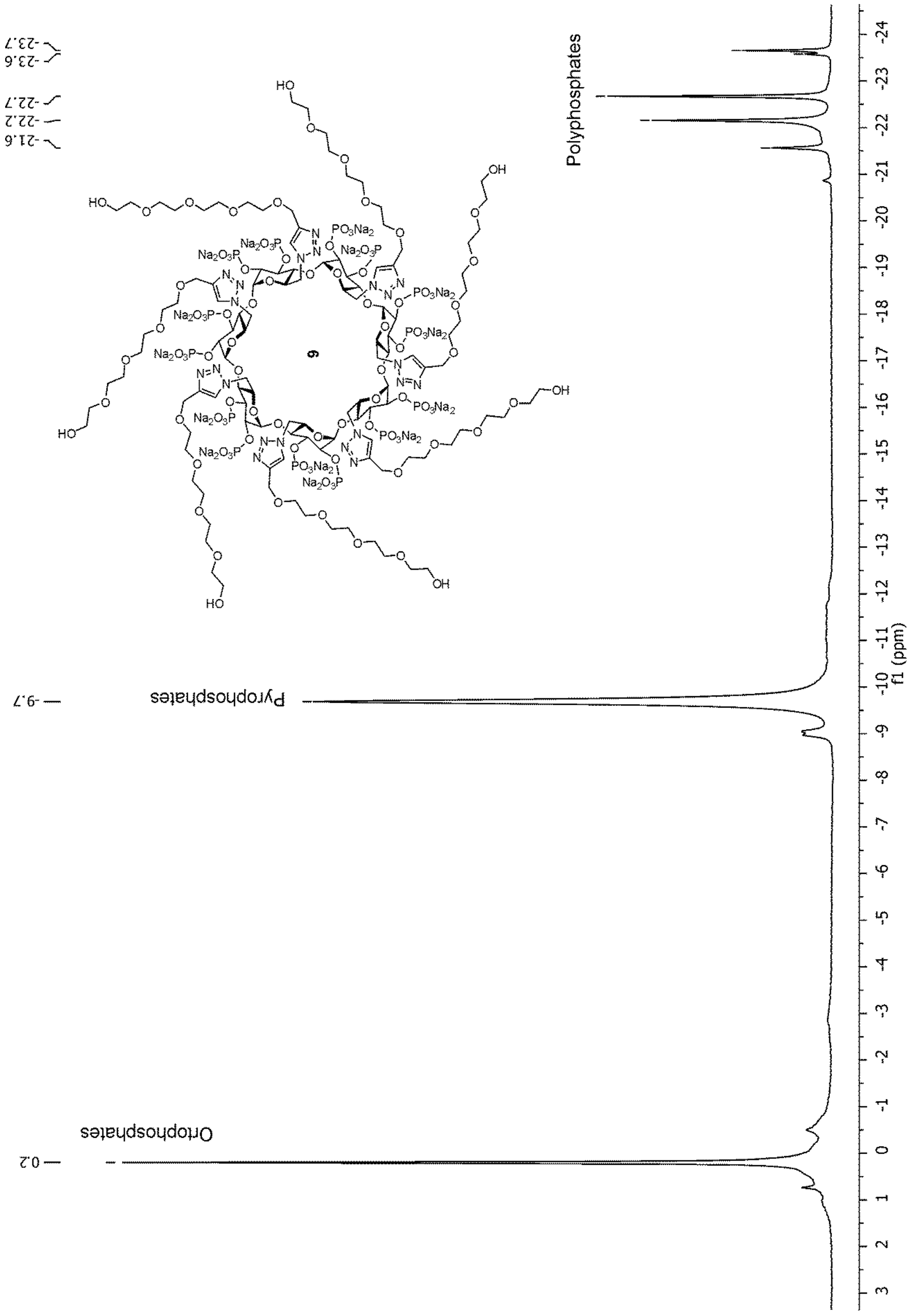

Figure S21. ^{31}P NMR spectrum (242.9 MHz, D_2O, 25 °C) for compound **9**

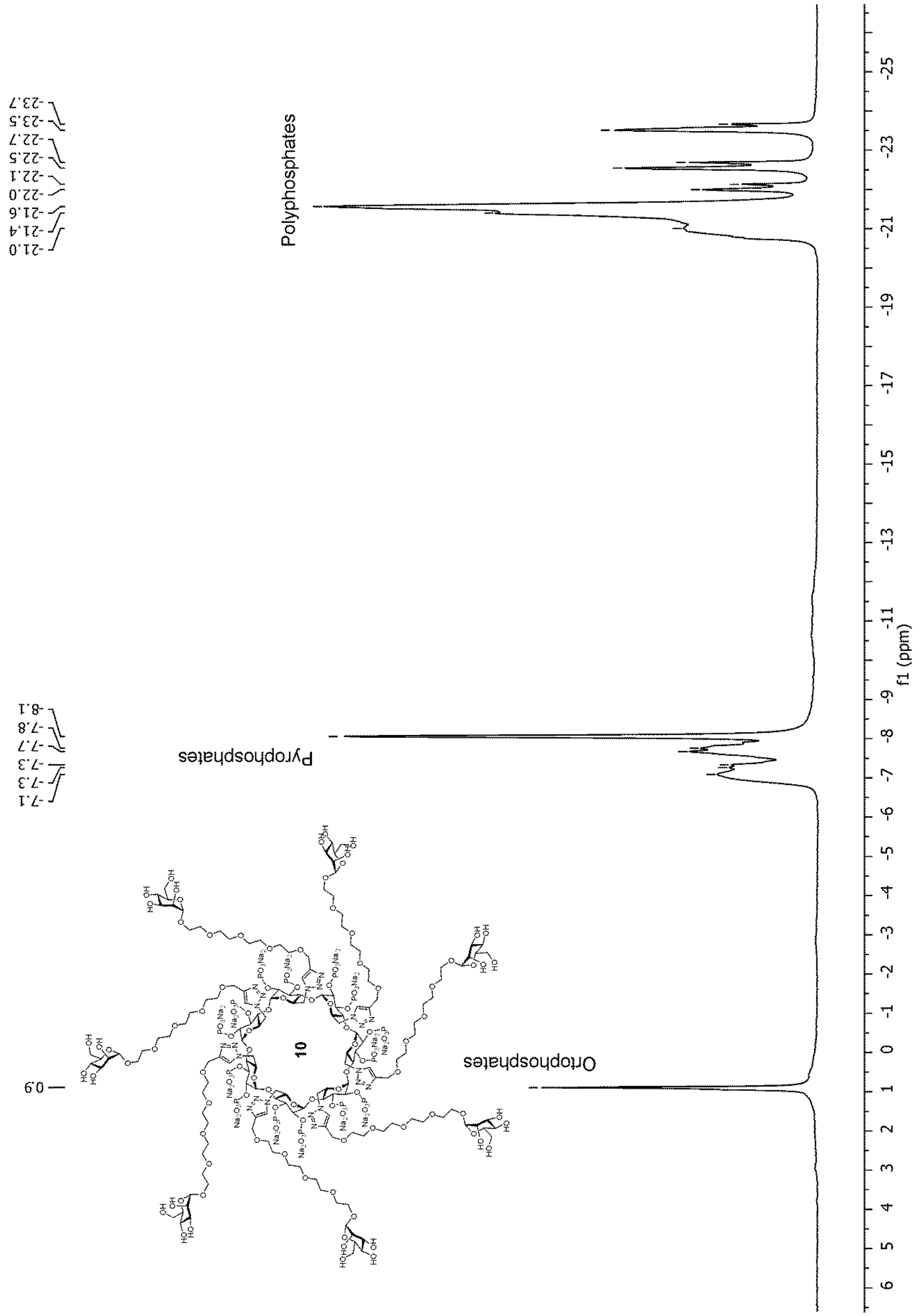

Figure S22. ^{31}P NMR spectrum (242.9 MHz, D_2O, 25 ºC) for compound **10**

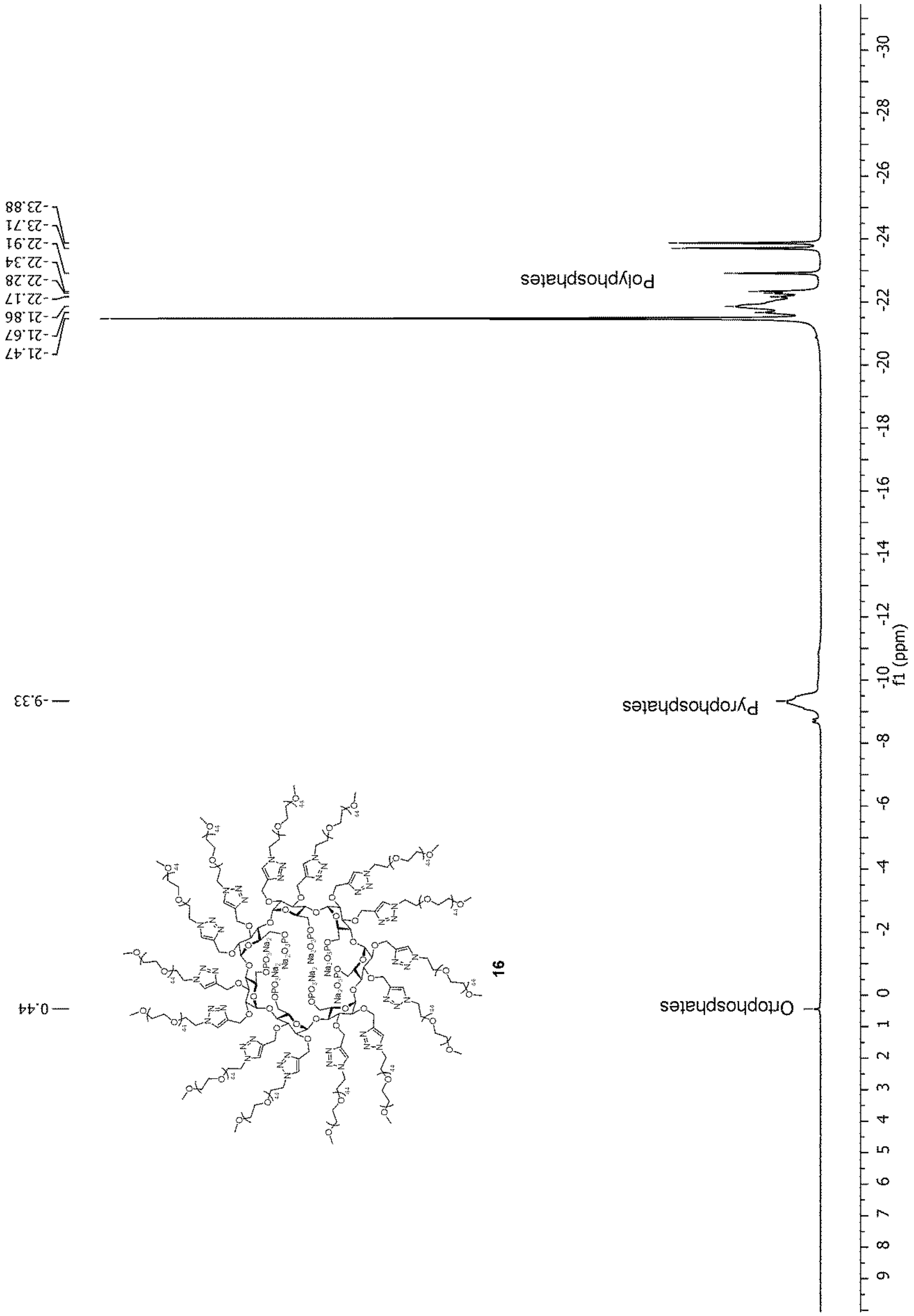

Figure S23. ^{31}P NMR spectrum (242.9 MHz, D_2O, 25 ºC) for compound **16**

IV. Determination of the number of phosphate groups for compounds 10-12 and 18

In order to estimate the number of phosphate groups presents on the structure of compounds **8-10** and **16**, we measured the %P w/w for each compound by HR-ICP-MS. Taking into account phosphorylation conditions that we have used during the synthesis, where an excess of P_2O_5 at 40 ºC was added, we assume that all free OH groups were phosphorylated in some extent to form orto-, pyro- and/or polyphosphate groups, according to the following reaction:

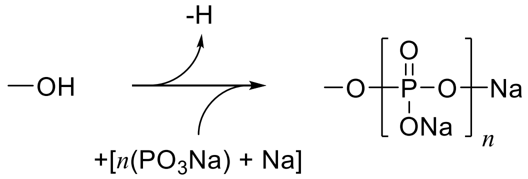

For a given molecule that contains phosphorus, we can define %P as:

$$\%\mathrm{P} = \frac{n\ x\ \mathrm{AW_p}}{\mathrm{MW}}\ x\ 100 \qquad (1)$$

where n is the number of atoms of P, AW_P is the atomic weight of P, and MW is the molecular weight of the phosphorylated molecule.

If we assume that all free OH groups were phosphorylated, for cyclodextrin derivatives **8-10** having 14 free OH groups on secondary face, equation (1) can be convert into:

$$\%\mathrm{P}(\mathbf{8\text{-}10}) = \frac{n\ x\ \mathrm{AW_p}}{\mathrm{MW}_{(\mathbf{8-10})} - 14\mathrm{H} + n(\mathrm{PO_3Na}) + 14\mathrm{Na}}\ x\ 100 \qquad (2)$$

where MW_X is the molecular weight of molecules **8-10**.

In the case of derivative **16** having only 7 free OH groups on the primary face, the equation (1) remains as:

$$\%\mathrm{P}(\mathbf{16}) = \frac{n\ x\ \mathrm{AW_p}}{\mathrm{MW}_{\mathbf{16}} - 7\mathrm{H} + n(\mathrm{PO_3Na}) + 7\mathrm{Na}}\ x\ 100 \qquad (3)$$

On the basis of equations (2) and (3), %P data obtained by HR-ICP-MS for compounds **8-10** and **16** allowed to estimate the following number of phosphate groups per molecule (n):

Table S1

Derivative	MW	%P (ICP)	Phosphate groups per molecule (n)	Average phosphate residues per free OH
**8**	2823.41	16.3	35.72	2.55
**9**	2921.91	15.7	34.03	2.43
**10**	4056.89	19.9	81.56	5.83
**16**	30212.48	8.0	106.49	15.21

V. MIL-100(Fe) nanoMOFs synthesis and surface modification

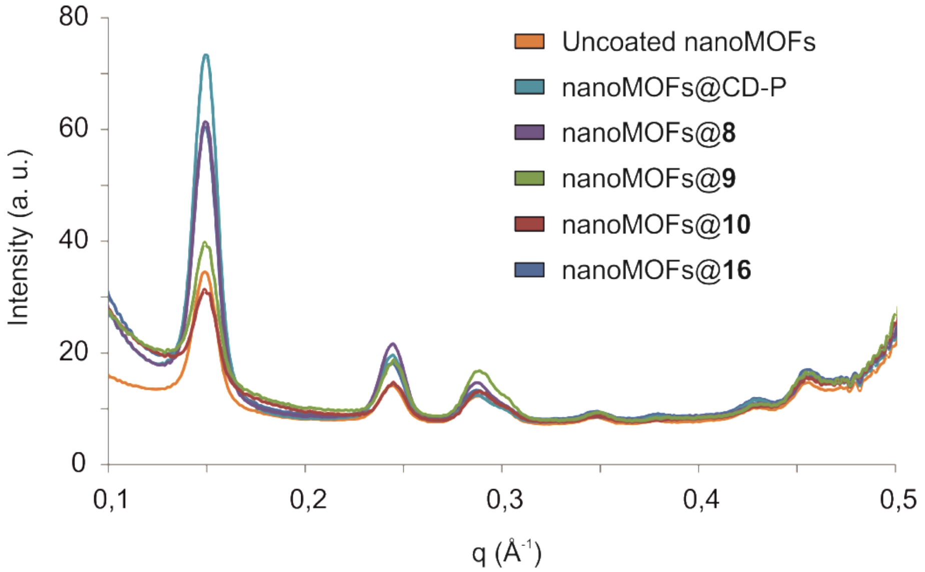

Figure S24. XRPD patterns of uncoated nanoMOFs and nanoMOFs coated with CD-P, **8-10** and **16**. To focus on the signal of the MOF lattice, only the range from 0.1 to 0.5 Å^{-1} is displayed.

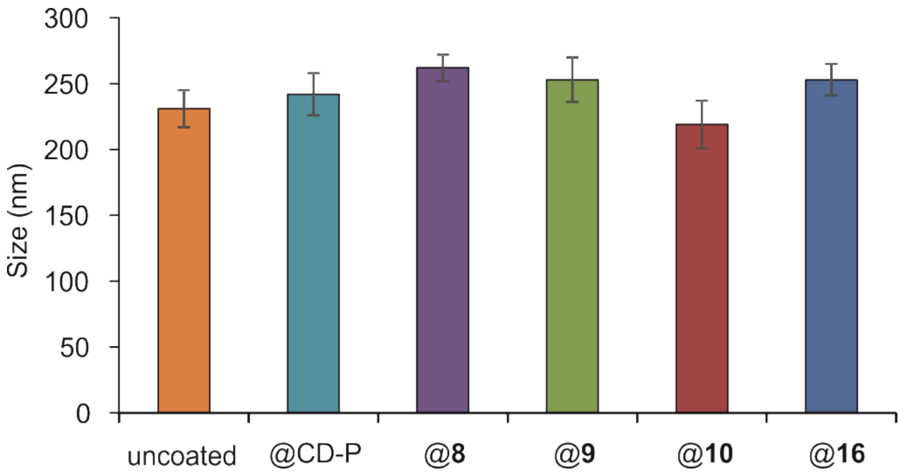

Figure S25. DLS size measurements of uncoated nanoMOFs and nanoMOFs coated with CD-P, **8-10** and **16** after 6 h incubation in DMEM cell culture medium complemented with 10% FBS.

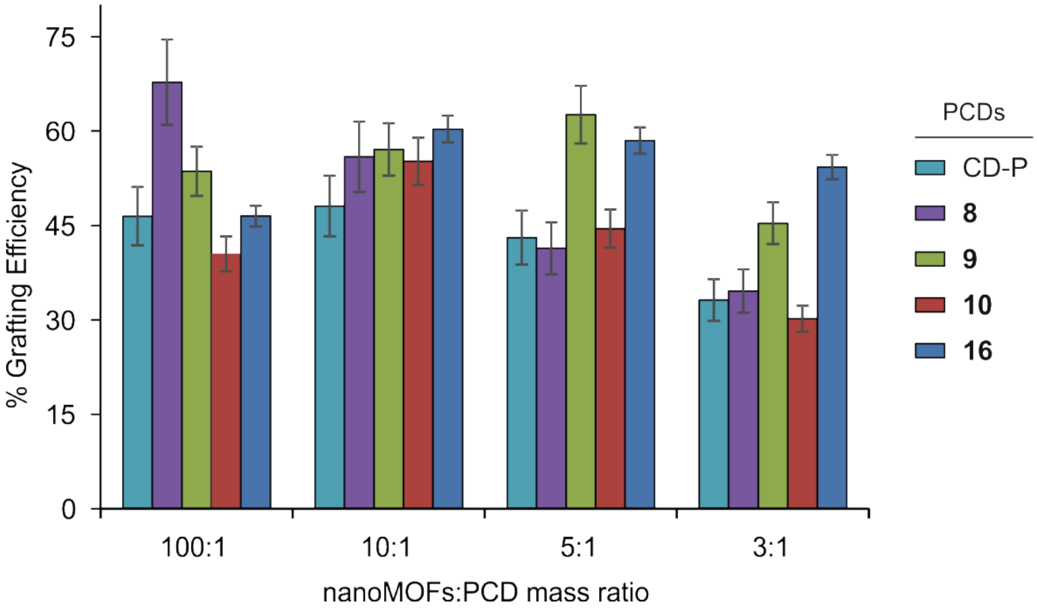

Figure S26. PCDs derivatives grafting efficiency versus nanoMOFs:PCD mass ratio assayed.

VI. ITC experiments for the interaction of 8, 10, nanoMOFs@8 and nanoMOFs@10 with Concanavalin A

Table S2

Conjugate	Buffer	[Conjugate]	[ConA][a] (μM)	Injection (μL)[b]
**8**	20 mM phosphate, pH 7.2	1.14 mM (syringe)	55.83 (cell)	8
**10**	20 mM phosphate, pH 7.2	1.29 mM (syringe)	33.61 (cell)	8
nanoMOFs@**8**	10 mM TRIS, pH 7.5	3.28 μM (cell)[c]	66.58 (syringe)	8
nanoMOFs@**10**	10 mM TRIS, pH 7.5	3.88 μM (cell)[c]	72.55 (syringe)	8

[a]Concentration of tetramer of ConA. [b]First injection was 2 μL in all cases. [c]Concentration of phosphates **8** and **10** anchored on the nanoMOFs surface considering an orthophosphate function per each OH group.

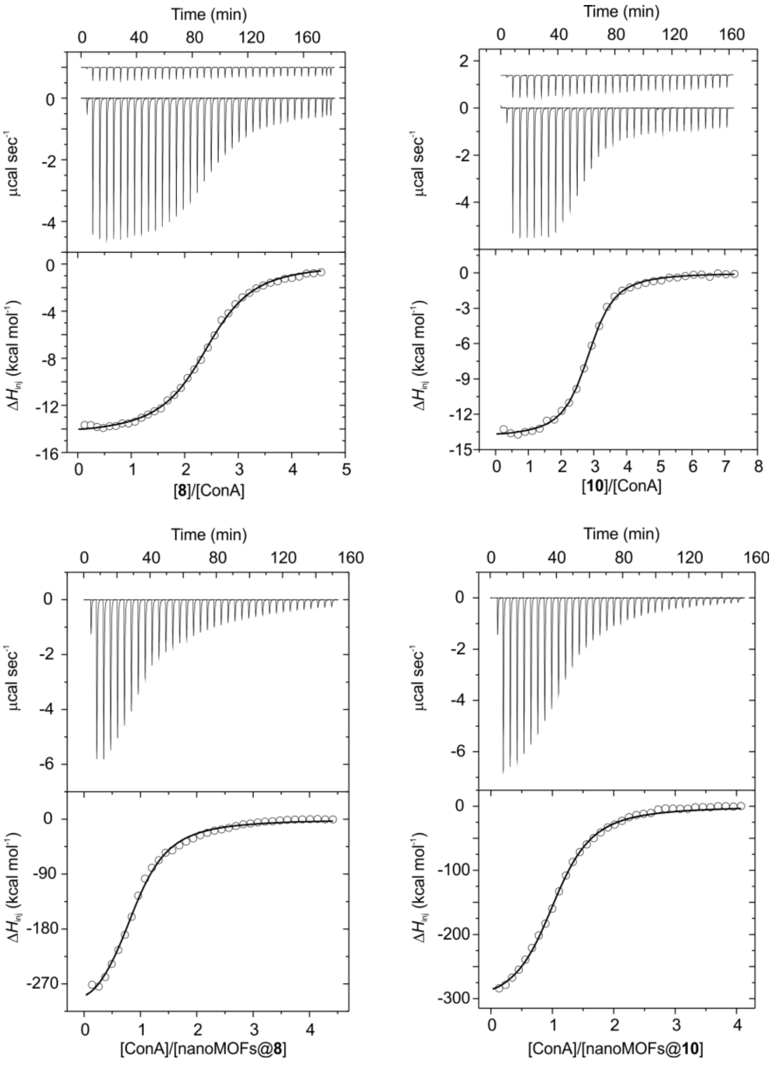

Figure S27. Titration of ConA with conjugates **8** (top, left) and **10** (top, right) in 20 mM phosphate buffer (pH 7.2) at 25 ºC, and titration of conjugates nanoMOFs@**8** (bottom, left) and nanoMOFs@**10** (bottom, right) with ConA in 10 mM TRIS buffer (pH 7.5) at 25 ºC. In all cases, the top panel shows the raw calorimetric data denoting the amount of exchanged heat following each injection of the titrating agent. The area under each peak represents the amount of heat released or absorbed upon binding of each conjugate to the lectin. Note that as the titrations progress the area under the peaks gradually becomes smaller because of the increasing saturation of the sugar binding sites of the protein. This area was integrated and plotted against the molar ratio of the titrating agent in syringe to the analyte in cell. The smooth solid lines represent the best fit of the experimental data to the model of *n* equal and independent binding sites.

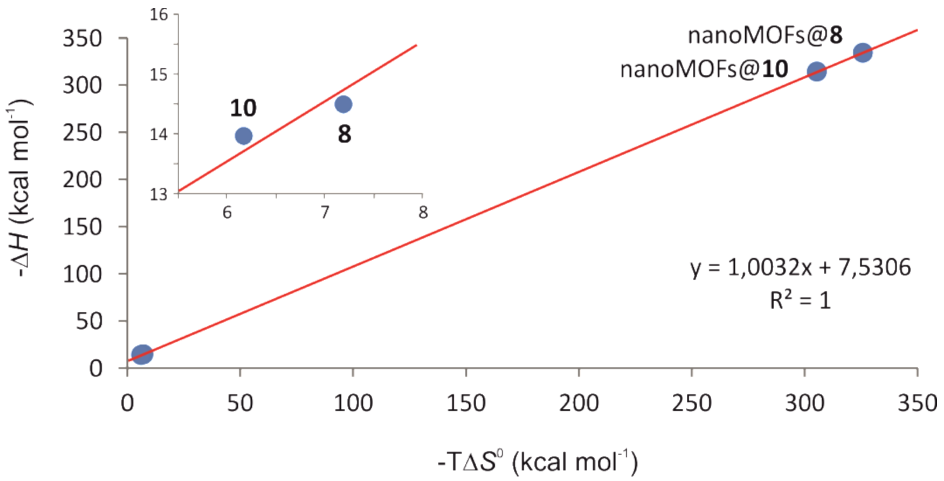

Figure S28. Enthalpy-entropy compensation for the interaction of **8**, **10**, nanoMOFs@**8** and nanoMOFs@**10** with ConA lectin at 25 ºC.
